# Structure of blood cell-specific tubulin and demonstration of dimer spacing compaction in a single protofilament

**DOI:** 10.1016/j.jbc.2024.108132

**Published:** 2024-12-24

**Authors:** Felipe Montecinos, Elif Eren, Norman R. Watts, Dan L. Sackett, Paul T. Wingfield

**Affiliations:** 1 Protein Expression Laboratory, National Institute of Arthritis and Musculoskeletal and Skin Diseases, National Institutes of Health, Bethesda, Maryland, USA; 2 Division of Basic and Translational Biophysics, Eunice Kennedy Shriver National Institute of Child Health and Human Development, National Institutes of Health, Bethesda, Maryland, USA

**Keywords:** tubulin, microtubule, cytoskeleton, blood, posttranslational modifications (PTM), cryptophycin, rings, compaction, interdimer, intradimer

## Abstract

Microtubule (MT) function plasticity originates from its composition of α- and β-tubulin isotypes and the posttranslational modifications of both subunits. Aspects such as MT assembly dynamics, structure, and anticancer drug binding can be modulated by αβ-tubulin heterogeneity. However, the exact molecular mechanism regulating these aspects is only partially understood. A recent insight is the discovery of expansion and compaction of the MT lattice, which can occur *via* fine modulation of dimer longitudinal spacing mediated by GTP hydrolysis, taxol binding, protein binding, or isotype composition. Here, we report the first structure of the blood cell-specific α1/β1-tubulin isolated from the marginal band of chicken erythrocytes (ChET) determined to a resolution of 3.2 Å by cryo-EM. We show that ChET rings induced with cryptophycin-52 (Cp-52) are smaller in diameter than HeLa cell line tubulin (HeLaT) rings induced with Cp-52 and composed of the same number of heterodimers. We observe compacted interdimer and intradimer curved protofilament interfaces, characterized by shorter distances between ChET subunits and accompanied by conformational changes in the β-tubulin subunit. The compacted ChET interdimer interface brings more residues near the Cp-52 binding site. We measured the Cp-52 apparent binding affinities of ChET and HeLaT by mass photometry, observing small differences, and detected the intermediates of the ring assembly reaction. These findings demonstrate that compaction/expansion of dimer spacing can occur in a single protofilament context and that the subtle structural differences between tubulin isotypes can modulate tubulin small molecule binding.

The microtubule (MT) cytoskeleton is involved in diverse processes such as chromosome partition, cellular motility, and intracellular transport, and therefore MTs are essential for cellular life. MT specialization to perform different functions involves the expression of tubulin isotypes with specific amino acid sequences, with the differences usually located in the C-terminal tails (1). In vertebrates, α- and β-tubulin isotypes are expressed from 8 and 9 genes, respectively, found in specific tissues, in different stages of development, and even in specialized structures within the same cell (2, 3). During the cell cycle, MTs are also subject to posttranslational modifications (PTMs) such as acetylation, detyrosination, phosphorylation, and glutamylation (1). The tubulin code hypothesis states that the expression of different tubulin isotypes and their PTMs modulate MT structure and function in the cell (1).

There are many examples of tubulin function modulation by isotype expression (4) and PTMs (5), but not all tubulin isotypes, nor PTMs, have been characterized. So far, the most well-characterized tubulin isotypes are the α-tubulins α1A and α1B, corresponding to the genes TUBA1A and TUBA1B; and the β-tubulins β2, β3, β4, and β5, corresponding to the genes TUBB2, TUBB3, TUBB4, and TUBB5 (previously referred to as TUBB or class I). In contrast, other less abundant and rarer tubulins are far less studied because they are harder to obtain. However, new technologies are being developed to study tubulin in all its forms through recombinant gene expression (6). An unusual case of monoisotypic tubulin that is naturally occurring is the blood cell-specific tubulin purified from the marginal band MTs of chicken erythrocytes (ChET) (7, 8, 9). This tubulin preparation comprises isotypes α1 and β1, corresponding to the genes TUBA1 and TUBB1 from *Gallus gallus*, which are orthologs of TUBA1A and TUBB1 from humans. TUBB1 expression is critical for platelet biogenesis (10), representing 50 to 90% of cytoplasmic β-tubulin, and defects in its function lead to congenital thrombocytopenia (11). Compared to other tubulin isotypes, ChET is nearly free of PTMs, and the only PTMs described for ChET are detyrosination of the α1-tubulin C-terminal tyrosine, the addition of taurine onto the detyrosinated α1-tubulin (9), and phosphorylation of the β1-tubulin at Ser441 (in only 10% of the isolated protein) (8). Therefore, ChET tubulin offers an opportunity to study tissue-specific and isotypically homogenous α1/β1-tubulin.

Previous cryo-EM studies revealed conformational changes in the MT lattice in response to external stimuli, most notably compaction or expansion of the longitudinal interface (reviewed in (12)). The axial repeat between tubulin heterodimers in the MT lattice (from now on termed as dimer spacing), is in the range 83.0 to 84.5 Å in expanded MT lattices and in the range 81.0 to 82.0 Å in compacted MT lattices (12, 13, 14). Therefore, the relative differences in dimer spacing between expanded and compacted MT lattices are only ∼1 to 3 Å. For instance, mammalian MTs undergo compaction of up to 1.2 Å upon GTP hydrolysis, while yeast MTs do not under the same experimental conditions (15). Another example is the binding of the MT end-binding protein 3 (EB3) that induces MT lattice compaction, although of only 0.7 Å (16). In the opposite case, induction of MT lattice expansion has been observed upon binding of taxol to GDP-MTs (14), or by binding of calmodulin-regulated spectrin-associated protein 3 (CAMSAP3) that can expand the MT lattice by up to 3% (17). Furthermore, studies have shown an effect of the αβ-tubulin isotype composition over the dimer spacing in the MT lattice. For example, the cryo-EM structure of MTs assembled from recombinantly expressed human tubulin isotypes α1A/β3, which are free of PTMs, showed altered polymerization (or interdimer) interfaces compared to the more heterogeneous brain MTs (18). Another example is the MT lattice compaction of MTs composed of different isotypes observed in PTM-free α4A/β2A MTs compared to α1C/β2A and α1A/β2A MTs, *i.e.*, where only the α-tubulin subunit is different (19). MTs composed of different tubulin isotypes may also undergo lattice expansion. For example, taxol has been shown to amplify the intrinsically different stabilities between *Danio rerio* α1/β4 and human α1/β3 MTs, inducing MT lattice expansion only in the former (20). This last study is important because the MT lattice expansion was coupled to effects on kinesin function, demonstrating possible *in vivo* significance. These findings support the idea that MT structure is modulated by the tubulin isotypes and PTMs it contains.

Recently, the cryo-EM structure of the anticancer drug cryptophycin-52 (Cp-52) bound to HeLa cell line tubulin (HeLaT-Cp-52) was solved by cryo-EM, revealing its mechanism of action (21). HeLaT includes the neural tubulin isotypes α1B, β3 (varying amounts), β4 (10–20%), and β5 (80–90%), and has PTMs such as phosphorylation and acetylation (22, 23, 24, 25). The addition of Cp-52 to HeLaT induces the formation of two sizes of rings composed of either eight or nine heterodimers that resemble those formed by the “peeling” of curved single protofilaments in a depolymerizing MT (except with tighter curvature) (26, 27). The HeLaT-Cp-52 ring is a single protofilament stabilized in the closed ring state, with curved interfaces at both tubulin interdimer and intradimer interfaces (21, 28).

In this study, we report the cryo-EM structure of the blood cell-specific tubulin isotype α1/β1 isolated from the marginal band of ChET solved to a resolution of 3.2 Å. We show that the addition of Cp-52 to ChET (ChET-Cp-52) induces the formation of rings that are smaller in diameter than HeLaT-Cp-52 rings composed of the same number of tubulin heterodimers. ChET intradimer and interdimer interfaces in Cp-52 rings appear compacted with shorter distances between tubulin subunits than HeLaT, accompanied by conformational changes mainly localized in the β-tubulin subunit. Furthermore, we detected a small difference in the Cp-52 apparent binding affinity between ChET and HeLaT, which may be related to the subtle conformational differences between the two tubulins. The observed shortening in ring diameter and compaction of interfaces is reminiscent of the compaction/expansion of dimer spacing in the MT lattice observed previously. These findings are discussed in the context of the role of tubulin isotypes over the MT lattice and protofilament structure.

## Results

### Cryo-EM structure of Cp-52-bound **α**1/**β**1−tubulin

**Figure 1:**
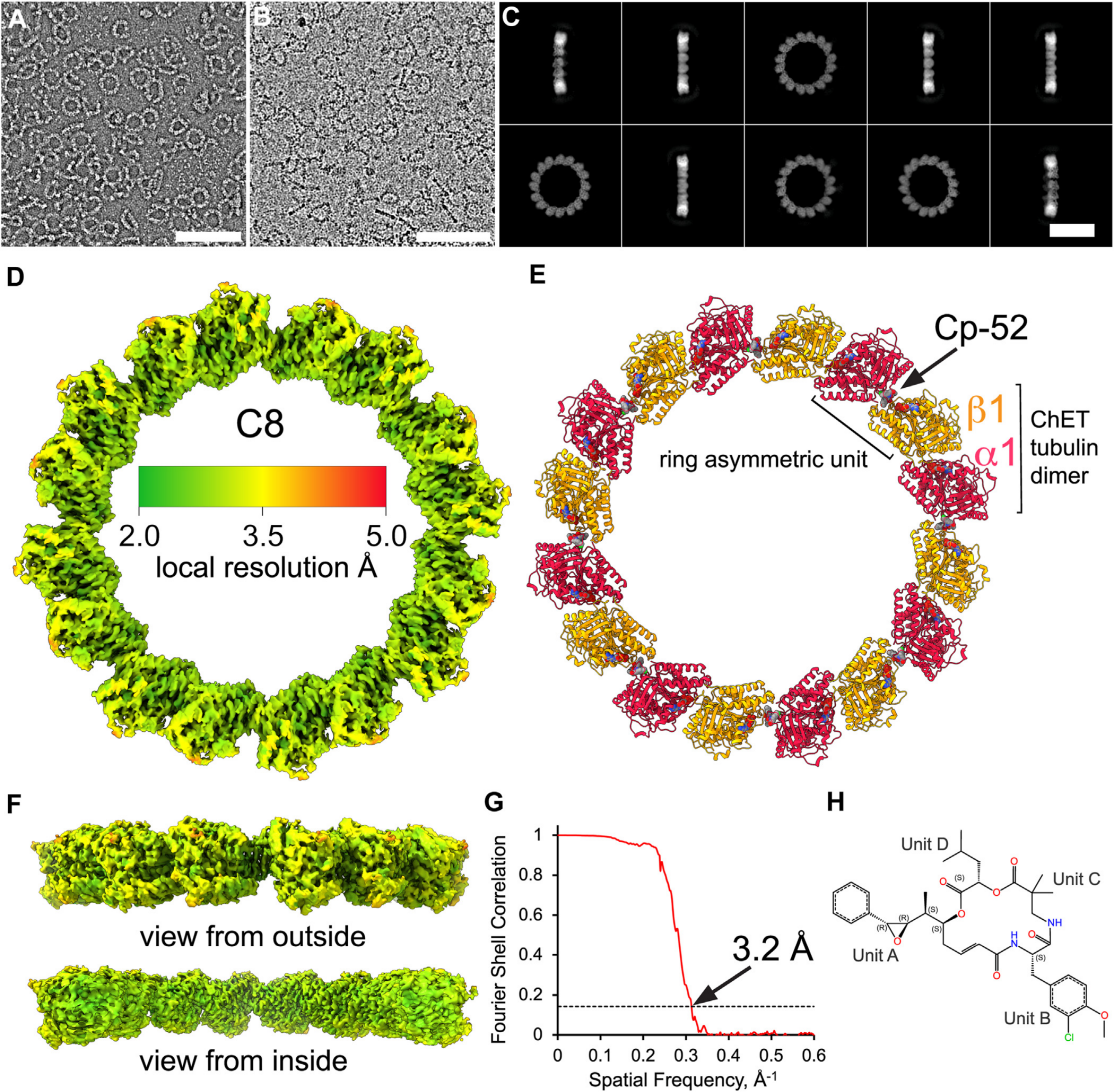
**Cryo-EM structure of α1/β1-tubulin bound to cryptophycin-52.***A*, electron micrograph of uranyl acetate-stained ChET α1/β1-tubulin rings induced by Cp-52 binding at room temperature. *B*, electron micrograph of vitrified ChET-Cp-52 rings. *C*, selected 2D classes obtained with CryoSPARC v4.3.1. Only rings with C8 symmetry (*i.e.*, eight ChET dimers) are shown. *D*, sharpened cryo-EM map colored according to local resolution estimation. *E*, ribbon representation of ChET-Cp-52 C8 structure with α1 in *red*, β1 in *orange*, and the molecules Cp-52, GDP, and GTP are represented in van der Waals radii representation. The *black brackets* indicate the position of the asymmetric unit of ChET-Cp-52, and of the canonical ChET α1/β1-tubulin heterodimer. *F*, views of the ChET-Cp-52 cryo-EM map from outside and inside of the ring. *G*, Fourier shell correlation curve for estimating the cryo-EM map resolution, resulting in a resolution FSC_0.143_ = 3.2 Å. *H*, 2D structure of Cp-52 bound to ChET obtained with the chemical sketch tool online (www.rcsb.org/chemical-sketch). The scale bars are A = 100 nm, B = 100 nm, C = 20 nm. ChET, chicken erythrocytes tubulin; Cp-52, cryptophycin-52.

To understand the role of isotype composition in the structure and conformation of αβ-tubulin, we solved the cryo-EM structure of tubulin purified from the marginal band of ChET, containing tubulin isotypes α1 and β1 expressed from the genes TUBA1A and TUBB1 (also called β tubulin class VI or βVI) (8). These genes from *Gallus gallus* are orthologs of *Homo sapiens* genes TUBA1A and TUBB1, and to date, the structure of TUBB1 has not been solved. To achieve this, we took advantage of the ability of Cp-52 binding to induce the formation of stable tubulin rings that are amenable to single-particle cryo-EM to solve the structure of α1/β1-tubulin (Fig. 1). When combined with equimolar concentrations of Cp-52, ChET dimers polymerized into rings that are almost indistinguishable from the Cp-52 tubulin rings formed by α1B/β3-tubulin isolated from cultured HeLa cells (21), as determined by negative stain electron microscopy (Fig. 1*A*) and by cryo-EM (Fig. 1*B*). In vitrified ice, ChET-Cp-52 rings have a diameter of ∼22 and ∼32 nm, corresponding to ring species with C8 and C9 symmetry, containing 8 and 9 α1/β1-tubulin heterodimers, respectively. We generated initial particle stacks of the two ring species by 2D classification (Figs. 1*C* and S1), *ab initio* reconstruction, and 3D classification in the CryoSPARC package (https://cryosparc.com/updates) (29). The best 3D class was refined by nonuniform refinement, resulting in cryo-EM density maps with resolution FSC_0.143_ = 3.2 Å for the C8 ring and 3.5 Å for the C9 ring, respectively (Figs. 1*G* and S1*E*). The local resolution calculation shows finer details on the inside of the ring than on the outside (Figs. 1, *D*–*F* and S1, *B–D*). A 2D representation of the Cp-52 structure as bound to ChET is shown in Figure 1*H*. Refined atomic models were obtained for both C8 and C9 maps, and the C8 model was selected for further analysis due to the better resolution of its density map.

The asymmetric unit of the ChET-Cp-52 C8 ring comprises one copy of α1-tubulin with its bound GTP, one copy of β1-tubulin with its bound GDP, and one molecule of Cp-52 between the tubulin subunits (Fig. 1*E*). The canonical ChET α1/β1-tubulin dimer is indicated on the outside of the ring atomic model in Figure 1*E*. Examination of the ChET-Cp-52 cryo-EM density map revealed resolutions ranging from ∼2.5 Å on the inside of the ring (green areas in Fig. 1*D*) to ∼4.5 Å on the outside of the ring (orange areas in Fig. 1*D*). This local resolution distribution is similar to what is observed on the related HeLaT-Cp-52 ring (21). To analyze the influence of sequence conservation and structural flexibility over the local resolution, we plotted the B-factor and sequence conservation index on the refined atomic model of the ChET-Cp-52 ring (Fig. S2). Tubulin loops in the outer regions of the ring show high flexibility (high B-factor) compared to the rest of the structure (Fig. S2*A*). These loops are the relatively unstructured H1-S2-loop (ChET aa. 23–64) and the M-loop (ChET aa. 276–285) of α- and β-tubulin, respectively (Fig. S2, *C* and *D*). The amino acid sequence conservation of the loops is different, with the M-loop showing higher conservation than the H1-S2-loop (Fig. S2*B*). The full αβ-tubulin sequences and the corresponding secondary structure assignments are shown in Fig. S3. The H1-S2-loop is rich in polar and charged residues and has many small side chain residues like glycine, alanine, leucine, and serine, especially in α-tubulin. These residues with small side chains contribute to the local flexibility of the H1-S2-loop, which is often unresolved in previous tubulin structures (30, 31). While the H1-S2-loop has not been associated with a specific function in tubulin, the M-loop has a crucial role in establishing lateral contacts in the MT lattice, undergoing robust conformational changes between the free dimer and polymerized state (32). Therefore, these two loops show an intrinsic flexibility that can explain the overall lower resolution of these regions located on the outside of the ring (corresponding to the MT lumen).

### Comparison of ChET-Cp-52 and HeLaT-Cp-52 ring structure

**Figure 2:**
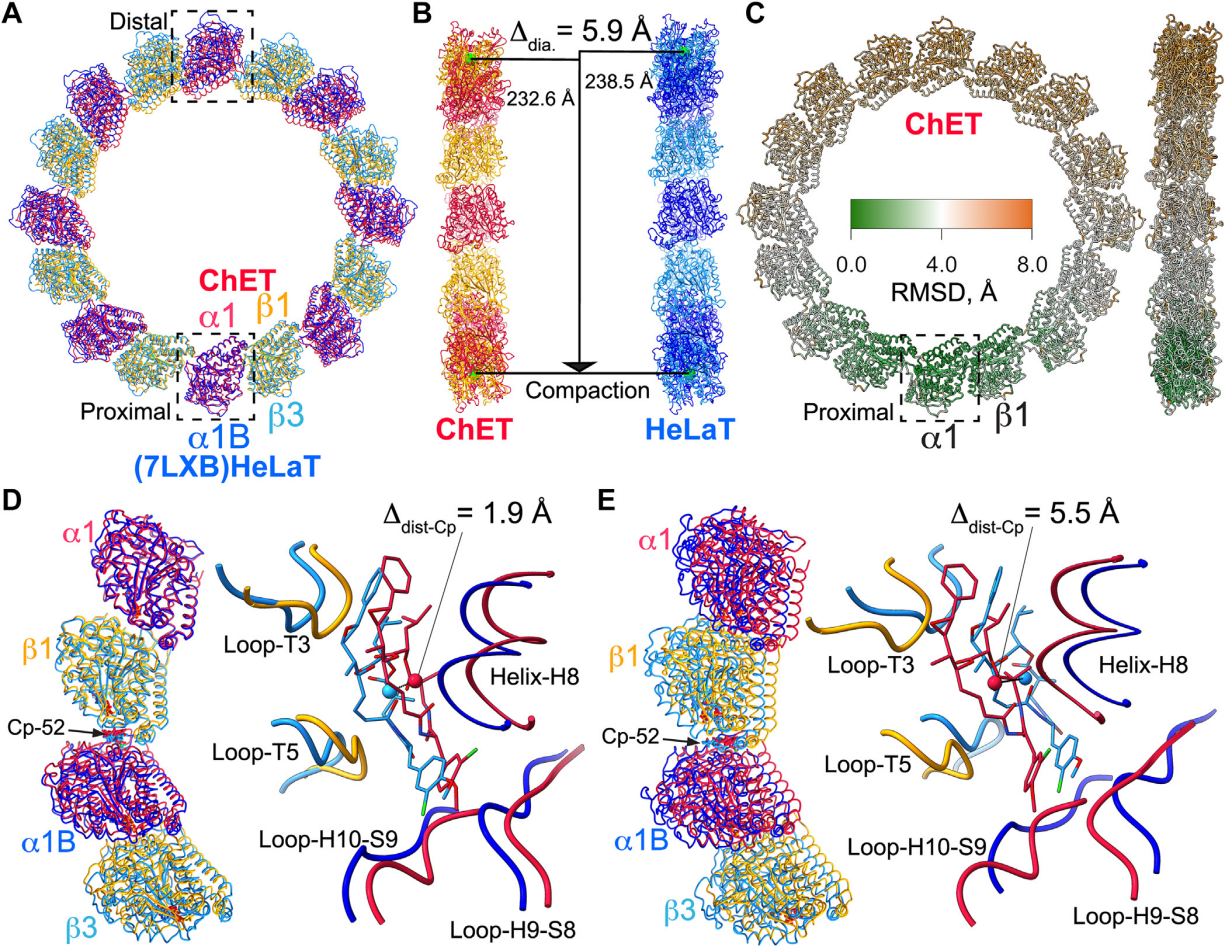
**Superposition of ChET-Cp-52 and HeLaT-Cp-52 C8 ring structures showing ChET ring compaction.***A*, the structures of ChET- and HeLaT-Cp-52 rings were structurally aligned using the α-tubulin proximal subunits (α1 for ChET and α1B for HeLaT, *dashed square* at the bottom). *B*, the diameters of the two Cp-52 rings measured between the centers-of-mass of their proximal and distal α-tubulin subunits indicate a 5.9 Å difference. *C*, representation of the gradual misalignment between ChET- and HeLaT-Cp-52 rings plotted as the RMSD over the ChET protein backbone. *D* and *E*, show the superposition of proximal and distal ChET and HeLaT tetramers and close-up views of Cp-52 binding sites. The center-of-mass distance between the Cp-52 molecules bound to each asymmetric unit is shown. Cp-52, cryptophycin-52; HeLaT, HeLa cell line tubulin.

We analyzed the ChET-Cp-52 ring structure to determine if the absence of isotype heterogeneity in ChET samples may translate into differences in the structure compared to HeLaT-Cp-52. We superimposed the two ring structures at their α-tubulin subunits because these proteins share the highest sequence identity, reaching 99.6% (subunit labeled “proximal” inside the dashed square in Figure 2*A*). A visual inspection of the structural superposition indicated that the two rings had a different diameter. Measurements of the ring diameter using the α-tubulin subunit center-of-mass showed that the ChET ring structure had a diameter 5.9 Å shorter than the HeLaT ring structure (Fig. 2*B*). This difference in ring diameter between the ChET and HeLaT rings is supported by the electron microscope magnification calibration (more details in Experimental procedures). Quantification of the protein backbone RMSD showed that the shifts are minimal (<1 Å RMSD) at the proximal side of the ring and maximal near the distal side of the ring (∼8 Å RMSD) (Fig. 2*C*). This compaction of the ChET ring compared to the HeLaT ring is reminiscent of the compaction observed in the cryo-EM structures of GDP-MT *versus* GTP (GMPCPP)-MT or of tubulin with different isotype composition observed previously (18, 19). The opposite, MT lattice expansion, has also been observed upon binding of taxol to GDP-MTs with different isotype composition (20). When considering an α1/β1-tubulin tetramer extracted from the aligned ring structures, we observed a gradually increasing shift in the position of the tubulin dimers along the ring (Fig. 2, *D* and *E*). For instance, the center-of-mass of the bound Cp-52 in the middle of the proximal tubulin tetramer is shifted by 1.9 Å between the two structures, while that of the distal tetramer is shifted by 5.5 Å. We also analyzed the α1/β1-tubulin intradimer and interdimer interfaces of the superimposed ChET-Cp-52 and HeLaT-Cp-52 rings, and the obtained structural parameters are presented in Table 1. Using a similar approach as for the comparison of ring diameters, we measured the distances between the tubulin subunits’ centers-of-mass for the intradimer and interdimer interfaces in the Cp-52 tubulin ring, observing that ChET subunits are positioned at shorter distances at both interfaces compared to HeLaT. Specifically, the intradimer interface is more compacted than the interdimer interface, with differences of 1.8 Å *versus* 0.6 Å, respectively (Table 1). These values are in the same range as the 1.2 Å compaction observed in cryo-EM structures of yeast MTs containing GTPγS *versus* GMPCPP (15). Furthermore, structural parameters such as the interaction surface area, number of residues, and number of bonds all indicate greater compaction of the intradimer interface than for the interdimer interfaces in ChET *versus* HeLaT (Table 1).

**Table 1:** Structural parameters of tubulin interfaces and of Cp-52 binding site

Structural property	ChET **α**1/**β**1-tubulin	HeLaT **α**1B/**β**3-tubulin
	Intradimer interface^a^	

Subunit center-of-mass distance^b^, Å	42.0	43.8
Number of residues	71	64
Area, Å^b^	1360	972
Number of bonds (HB+SB+DS)	14	7

	Interdimer interface^a^	

Subunit center-of-mass distance^a^, Å	48.1	48.7
Number of residues	27	29
Area, Å^b^	513	519
Number of bonds (HB+SB+DS)	7	1

	Cp-52 binding site^c^	

Number of residues	24	17
Area, Å^b^	2839	2606

DS, disulfide bonds; HB, hydrogen bonds; SB, salt bridges

a Interface area, number of residues, and number of bonds obtained with ePISA.

b Measured using the center-of-mass of each subunit in ChimeraX.

c Number of residues and area calculated in ChimeraX with the commands “zone” and “measure sasa”, respectively.

Values for MT dimer spacing in the literature range from 81.0 to 84.5 Å, depending on the experimental conditions (12, 13, 16). Comparing the dimer spacing from the Cp-52 tubulin ring to the literature values is difficult because the literature values refer to dimer spacing in straight MT lattices that include longitudinal and lateral interactions. In contrast, the dimer interfaces of the Cp-52 tubulin ring structure are curved and correspond to a single protofilament, *i.e.*, in the absence of lateral interactions. Additionally, the circular geometry of the rings means that points on adjacent dimers that are at the periphery of the ring (outer circumference) will necessarily be farther apart than ones at the interior of the ring (inner circumference). To approach this, we structurally aligned the Cp-52 tubulin ring and the GDP-MT (6DPV), through their α-tubulin subunits, and looked for the point-of-contact (POC) of the longitudinal (interdimer) interface that overlaps between the two structures (Fig. S4). For HeLaT- and ChET-Cp-52 rings, the residues α:P263 and β:H396 are closest to the GDP-MT residues α:P263 and β:H406, respectively, and both are located at the longitudinal interface. Using this POC point near the ring's inner circumference (at the MT outside), we obtain the diameter, D_POC_, as the distance between POCs on proximal and distal α-tubulin subunits (illustrated in Fig. S4). This yields D_POC_ = 209.2 Å for HeLaT and D_POC_ = 204.0 Å for ChET. Then, the dimer spacing for the Cp-52 tubulin ring containing eight dimers is obtained by the formula Dπ/8, yielding 82.2 Å spacing for HeLaT-Cp-52, and 80.1 Å for ChET-Cp-52. This compares to 81.6 Å dimer spacing for GDP-MT measured using the POC residues defined in Fig. S4. As a reference, the equivalent distance previously measured as dimer rise (similar to dimer spacing here) is 81.7 Å (16). The difference in the dimer spacing between HeLaT- and ChET-Cp-52 rings is 2.1 Å, essentially the same as the shift in spacing previously observed for GTP (GMPCPP) MT and GDP-MT (12, 13, 14). For these reasons, we take the HeLaT-Cp-52 ring as our reference structure and refer to the differences observed in the ChET-Cp-52 ring as due to compaction of the longitudinal interface.

### ChET-Cp-52 conformational changes

The αβ-tubulin dimer can be found to favor a curved conformation when free in solution or a straight conformation when polymerized into MTs, and the structures of both conformations have been solved experimentally (reviewed in (33)). Typically, these conformational transitions of tubulin and other members of the tubulin-FtsZ superfamily upon polymerization include rotations and translations between the N- and C-terminal domains, with H7-helix functioning as a hinge (33, 34). As previously observed, HeLaT-Cp-52 shows curvature localized at the intradimer and the interdimer interfaces (21, 28). The ChET-Cp-52 C8 ring structure presented here shows compacted tubulin interfaces, causing it to have a smaller diameter than the HeLaT-Cp-52 C8 ring. To quantify the conformational changes occurring in the ChET dimer, we structurally aligned it with the HeLaT-Cp-52 tubulin dimer through their α-tubulin subunits (Fig. S5) and measured structural parameters following the method introduced by Knossow *et al.* (33). The structure of the ChET α1/β1-tubulin dimer is shown in Figure 1*E* (label outside of the ring), comprising one α1-tubulin and one β1-tubulin with bound GTP and GDP, respectively. Visual inspection of the β1-tubulin subunit from the aligned tubulin dimers indicates that the bending at the intradimer interface is not identical between ChET (red structure in Fig. S5) and HeLaT (blue structure in Fig. S5). Still, both show a curvature that is intermediate between the curved (Protein Data Bank (PDB) ID: 6GWC) and straight (PDB ID: 6DPU) tubulin dimers. We measured the angle between the H7-helix of the aligned dimers and the geometric transformation matrix required to align the β-tubulin subunits, which includes RMSD, rotation, and translation (summarized in Fig. S5). These additional measures confirm that the ChET-Cp-52 structure is more closely aligned to curved tubulin than straight tubulin.

**Figure 3:**
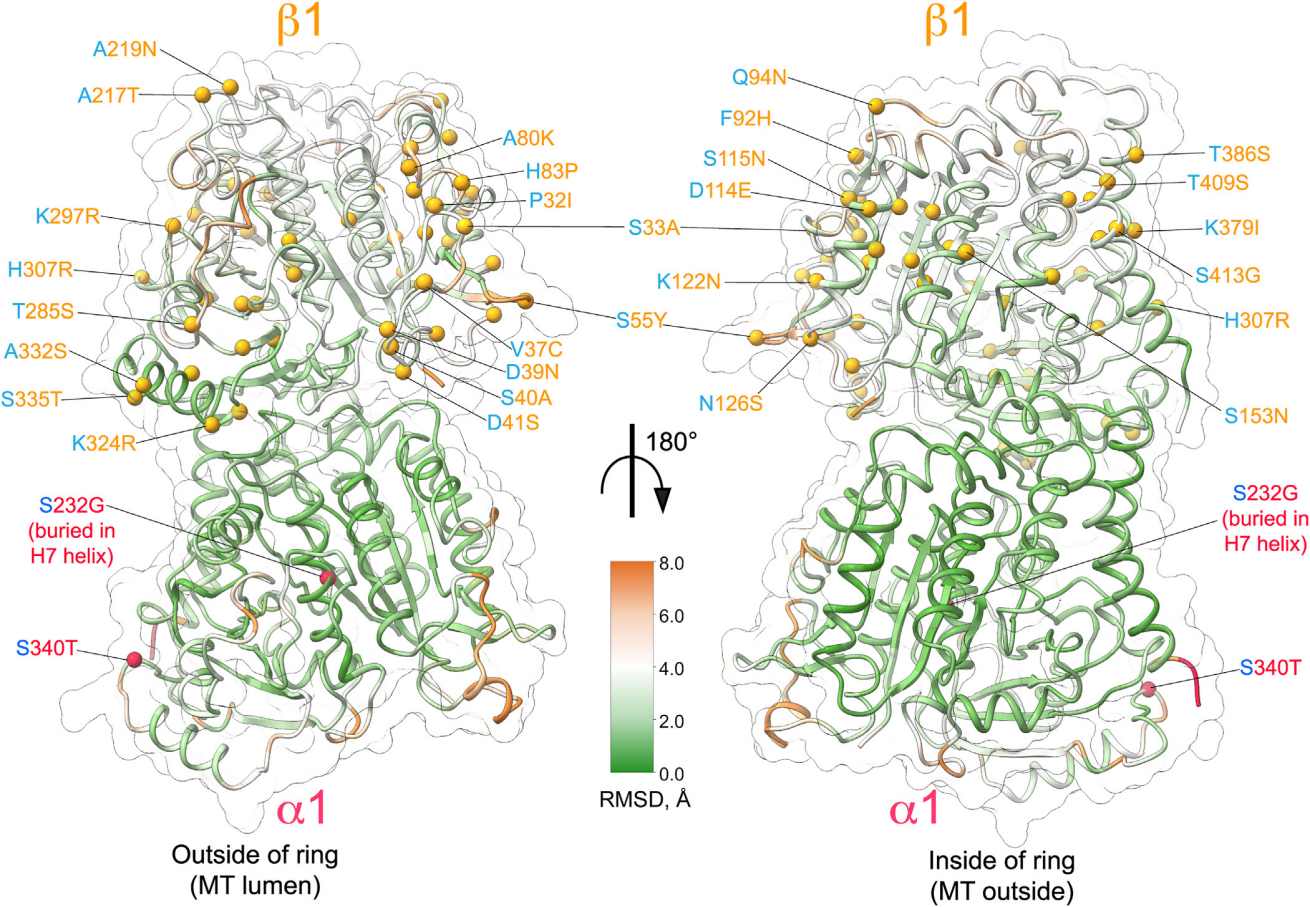
**ChET-Cp-52 α1/β1-tubulin heterodimer backbone RMSD and residue substitutions compared to HeLaT-Cp-52.** The RMSD between ChET and HeLaT αβ-tubulin heterodimers, following structural alignment at their α-subunits, is represented by the coloring of the ChET backbone. Views are from the MT lumen (*left*) and MT outside (*right*), respectively. The amino acid substitutions in ChET compared to HeLaT are marked over the backbone of the ChET heterodimer, with two substitutions in ChET α1-tubulin (*red spheres*) and 53 in β1-tubulin (*orange spheres*). Only the substitutions located at the surface of the αβ-tubulin heterodimer are labeled. ChET, chicken erythrocytes tubulin; Cp-52, cryptophycin-52; HeLaT, HeLa cell line tubulin; MT, microtubule.

To further compare the conformational changes between the aligned ChET-Cp-52 and HeLaT-Cp-52 αβ-tubulin dimers, we measured the backbone RMSD between them and mapped the residue substitutions over the aligned structure (Fig. 3). The diagram shows that the conformational changes are predominantly localized to the core of the β-tubulin subunit with an RMSD in the range of 3 to 5 Å. In contrast, the α-tubulin subunit has an RMSD ≤ 2 Å. Not surprisingly, most of the residue substitutions between ChET and HeLaT are found in β-tubulin with 53 substitutions, while only two changes are observed in α-tubulin (Figs. 3 and S3). Since many of these substitutions are located at the surface of the ChET α1/β1-tubulin dimer (Fig. 3), we estimated the effects of these changes over the structure's surface. We calculated the electrostatic and hydrophobicity potentials and compared them to the HeLaT potentials (Fig. S6). Visual inspection revealed that several patches are indeed different between the two proteins (marked by the dashed ovals) and that these patches are often localized to areas of the surface that are found on the outside of the Cp-52 tubulin ring, corresponding to the MT lumen, and on the inside of the Cp-52 tubulin ring, corresponding to the MT outer surfaces. Some patches located on the sides of the Cp-52 tubulin ring are also different, corresponding to the lateral interfaces of the MT. These changes will likely affect the properties of MT surfaces, which are sites of interactions with MAPs and other MT-interacting proteins that modulate MT stability and function (12).

### Structure and conservation of the ChET Cp-52 binding site

**Figure 4:**
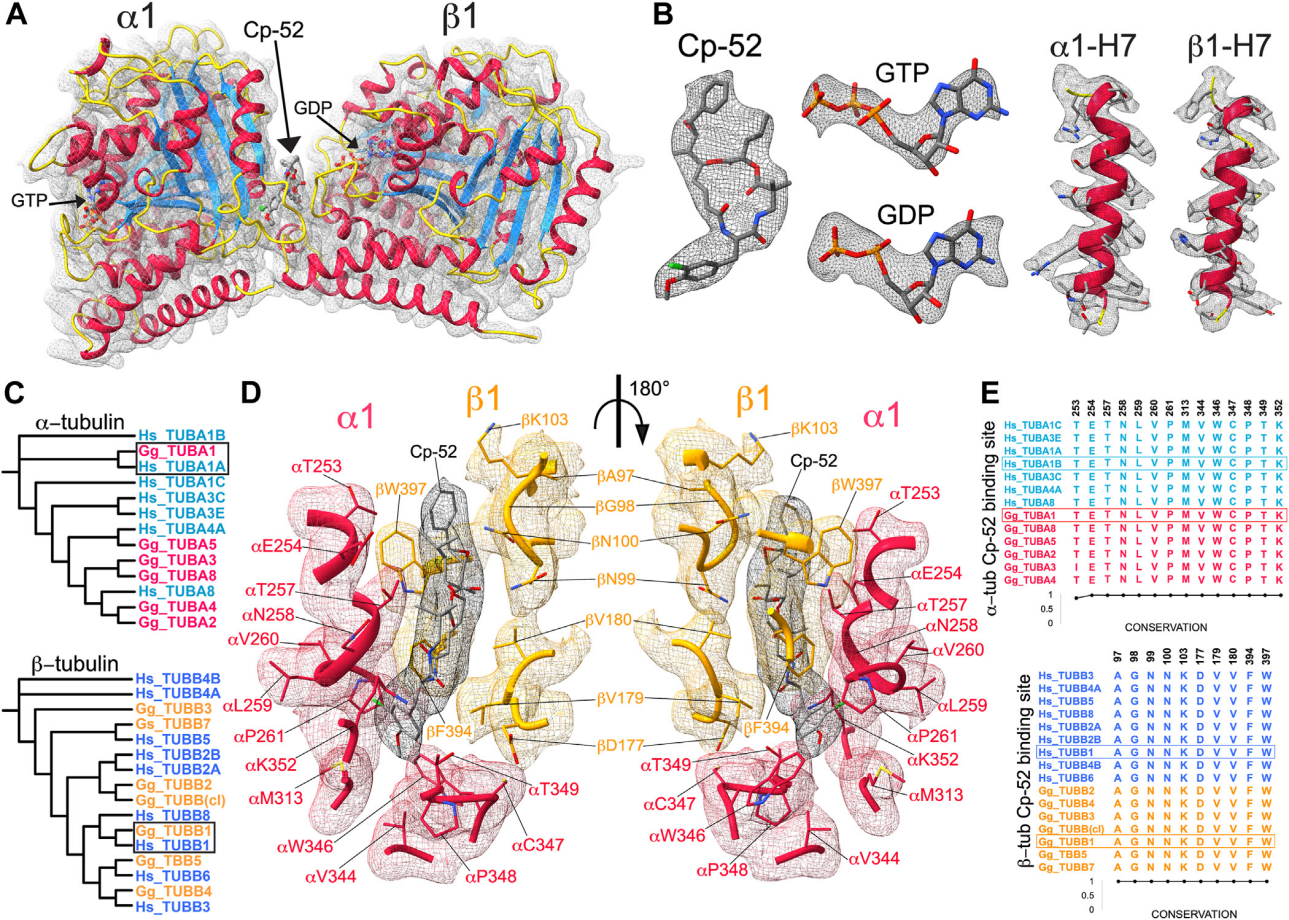
**ChET structure, cryo-EM density, and Cp-52 binding site amino acid conservation.***A*, ChET-Cp-52 asymmetric unit atomic structure and cryo-EM density (mesh representation). The ChET subunits α1 and β1 are colored according to secondary structure (*helices* are *red*, *strands* are *blue*, and *loops* are *yellow*). The positions of Cp-52, GDP, and GTP are indicated by *arrows*. *B*, close-up views of densities (mesh representation) for Cp-52, GTP, GDP, and the H7-helix from α1- and β1-tubulin, respectively. *C*, phylogenetic tree of α/β subfamilies showing the clustering of α- and β-tubulins from human (*Homo sapiens*) and chicken (*Gallus gallus*), enclosed by a *black box*. *D*, detailed view of the ChET-Cp-52 binding site cryo-EM density. The residues within 4 Å from the bound Cp-52 are shown, with α1 (Gg_TUBA1, *red*), β1 (Gg_TUBB1, *orange*), and Cp-52 (*gray*). *E*, amino acid sequence alignment and conservation of ChET-Cp-52 binding site residues. The conservation index was calculated as the reciprocal of the normalized Shannon entropy (using 20 as the number of amino acid types). ChET, chicken erythrocytes tubulin; Cp-52, cryptophycin-52.

We analyzed the structure and conservation of the ChET Cp-52 binding site in the interdimer interface to determine if the observed differences in structure and conformation may translate into differences in Cp-52 binding. First, we describe the binding site in ChET-Cp-52 complexes. A detailed view of the atomic structure and the cryo-EM density map corresponding to the ChET-Cp-52 asymmetric unit is shown in Figure 4*A*. The masked densities of the Cp-52, GTP, and GDP molecules are shown in Figure 4*B*. The refined map provided enough detail at ∼3 Å (or better) to fit the protein backbone and to resolve the H7-helix and its residues with bulky or long side chains (35). When considering the evolutionary sequence conservation of the α- and β-tubulin subfamilies from *Homo sapiens* and *Gallus gallus*, the ChET TUBA1A and TUBB1 translated gene sequences are grouped with human isotypes genes TUBA1A and TUBB1, respectively (Fig. 4*C*). So far, the α-tubulin gene TUBA1A (α1A) is the isotype most frequently encountered among the solved tubulin structures in the PDB database, while the structure of the β-tubulin gene TUBB1 (β1) has not been solved experimentally. Figure 4*D* shows the structure and densities of the Cp-52 binding site residues located within 4 Å from the bound Cp-52 molecule. When considering the tubulin isotypes from α- and β-tubulin subfamilies, all residues in the binding site are identical, with one exception: *Gallus gallus* TUBA3 gene that has isoleucine in position 253 instead of the conserved threonine in all the remaining sequences (Fig. 4*E*). This high sequence conservation means Cp-52 binding may not discriminate among tissue-specific tubulins.

**Figure 5:**
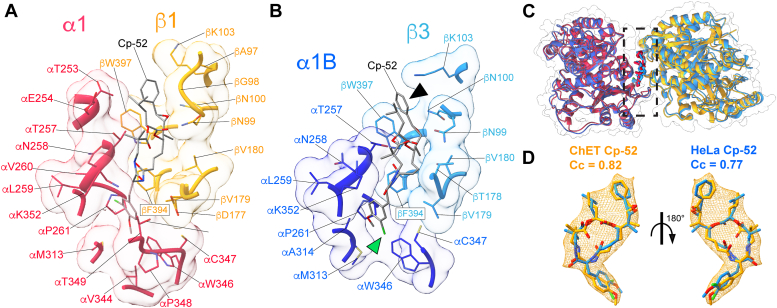
**Side-by-side comparison of ChET and HeLaT Cp-52 binding sites.** The residues within 4 Å of Cp-52 are displayed along with their solvent-accessible surfaces. The binding sites of ChET-Cp-52 (*A*) and HeLaT-Cp-52 (*B*), consist of 24 and 17 residues, respectively. The *green* and *black arrowheads* point to the two aromatic rings in Cp-52 that appear with a rotated orientation between the two structures. *C*, the structural superposition used to generate the images of the binding site (*dashed area*) in *A* and *B*. *D*, the structural superposition of the Cp-52 molecules obtained from each structure and fitted to the ChET-Cp-52 cryo-EM density (mesh representation in *orange*). ChET: chicken erythrocytes tubulin; Cp-52, cryptophycin-52; HeLaT, HeLa cell line tubulin.

We then compared the atomic structures of the Cp-52 binding site between ChET- and HeLaT-Cp-52 complexes (Fig. 5). Since there is a compaction of tubulin interfaces between these two ring structures, the number of residues and interface areas measured are greater for ChET than for HeLaT (Table 1). Although more residues are observed within 4 Å of the bound Cp-52 for ChET than for HeLaT, the overall arrangement of the binding site looks similar. This is not surprising since there is a total conservation of the participating residues. The Cp-52 structure can be divided into four units, A to D (21) (Fig. 1*H*). Notably, the bound Cp-52 shows differences in units A and B orientation between ChET and HeLaT structures. For instance, ChET Cp-52 unit A is rotated by ∼90° (black arrowheads in Fig. 5*B*). Also, unit B is rotated by 180° with the chlorine atom facing toward P261 in the ChET-Cp-52 structure while facing in the opposite direction (away from P261) in the HeLaT-Cp-52 structure (green arrowhead in Fig. 5*B*). These differences in the orientation of the bound Cp-52 between ChET and HeLaT suggest that the Cp-52 may bind with more than one pose to tubulin, possibly indicating conformational promiscuity (see Discussion). To determine if the Cp-52 is adopting two different conformations between ChET and HeLaT, we measured the RMSD between these structures and the cross-correlation when fitting to the ChET cryo-EM density map (Fig. 5*D*), resulting in RMSD = 1.57 Å, and Cc = 0.82 for the ChET conformation *versus* Cc = 0.78 for the HeLaT conformation. These numbers indicate that Cp-52 adopts a different conformation when bound to ChET rather than to HeLaT (36). Moreover, this structure of Cp-52 bound to ChET confirms our earlier observation that unit D of cryptophycin remains an optimal point for linker attachment due to its orientation facing away from the binding site (21). Recently, this aspect has been exploited for designing cytotoxic antibody-drug conjugates for targeted delivery into selected cells, where the Cp-52 molecule is attached to the cell-penetrating peptide through its D-unit (37, 38).

### Determination of Cp-52 apparent binding affinity

**Figure 6:**
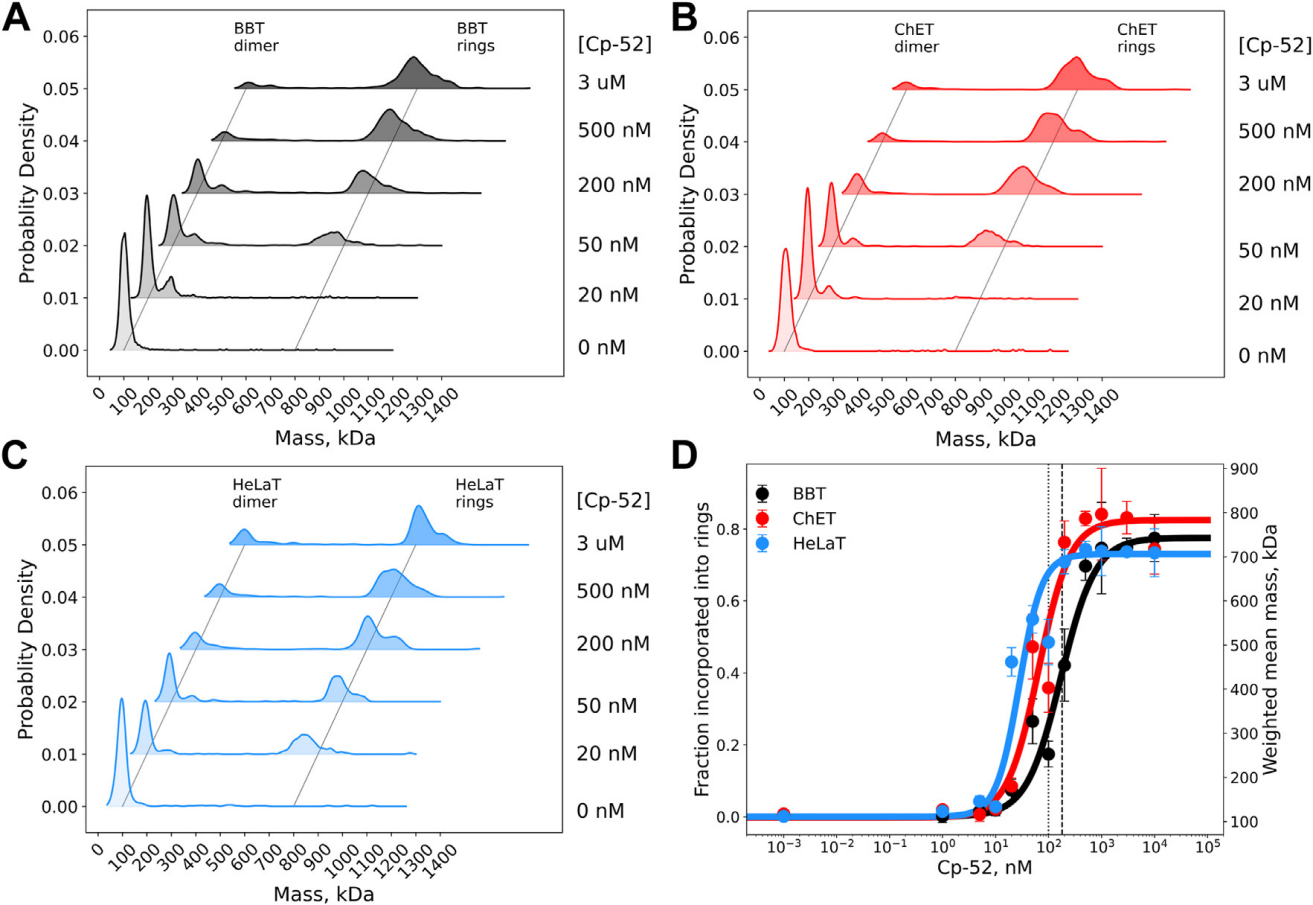
**Quantification of the tubulin Cp-52 apparent binding affinity by mass photometry.***A*–*C*, mass photometry kernel density distribution as a function of Cp-52 concentration. The identified peaks corresponding to the αβ-tubulin heterodimer (∼100 kDa) and the Cp-52 rings (700–900 kDa) are indicated by the *straight lines*. *D*, Cp-52 binding isotherms of bovine brain tubulin (BBT), ChET, and HeLaT obtained from the mass kernel density distributions shown in panels *A*, *B*, and *C*. The error bars are the standard deviations of replicate measurements in each condition. The isotherms were fitted to Equation 1, and the parameters are shown in Table 2. The *vertical lines* indicate the K_d_ values for Cp-52 binding reported previously in the literature: 97 nM (*dotted line*) (48) and 277 nM (*dashed line*) (53). ChET, chicken erythrocytes tubulin; Cp-52, cryptophycin-52; HeLaT, HeLa cell line tubulin.

To quantify and compare the interactions of cryptophycin with ChET and HeLaT, we characterized their assembly into rings upon Cp-52 binding using mass photometry and estimated the Cp-52 apparent dissociation constant (K_d-app_) from these data (Fig. 6). To test the suitability of mass photometry for detecting the assembly of tubulin-Cp-52 rings, we first characterized bovine brain tubulin (BBT). In the absence of Cp-52, the mass photometry kernel density distribution of BBT showed a single species with a mean mass of 104 ± 22 kDa, corresponding to the BBT dimer (1X, X = BBT dimer) (0 nM Cp-52, Fig. 6*A*). Addition of Cp-52 in the range of 1 to 20 nM induced the formation of a species at ∼200 kDa corresponding to a BBT tetramer (2X) (Fig. 6*A*). At 50 to 200 nM Cp-52, the distribution revealed the presence of BBT dimers (1X), tetramers (2X), and traces of a species in the range of 700 to 800 kDa corresponding to the tubulin rings (∼8X) (Fig. 6*A*). Still, at 50 to 200 nM Cp-52, the BBT dimer is the main species detected. In the range of 0.5 to 3 μM Cp-52, the tubulin ring species at 700 to 900 kDa (∼8X) are the main species, surpassing the BBT dimer population (Fig. 6*A*). Notably, at 3 μM Cp-52, the distribution shows sparsely populated intermediate species (in the range of 200–600 kDa) that could not be resolved clearly but consistently appeared in replicate experiments. To resolve these peaks, we lowered the BBT concentration by 10-fold, and at 100 nM Cp-52, the mass photometry distribution resolved multiple peaks corresponding to 1X, 2X, 3X, and 4X (*i.e.*, BBT dimers, tetramers, hexamers, and octamers), and the 8X and 9X species corresponding to the tubulin Cp-52 rings with C8 and C9 symmetry that we have previously resolved using cryo-EM (Fig. S8*A*). The C8 and C9 Cp-52 rings are the main polymeric species in these samples with saturating Cp-52 concentrations. The mass photometry experiments with ChET (Fig. 6*B*) and HeLaT (Fig. 6*C*) revealed a behavior similar to that of BBT. In the absence of Cp-52, the estimated molecular mass of ChET dimers is 106 ± 19 kDa and that of HeLaT dimers is 105 ± 33 kDa. In the range of 50 to 200 nM Cp-52, the 2X species (tetramer) appeared, and at ∼50 nM Cp-52, the ring species (7X–8X) could be detected. At 0.5 to 3 μM Cp-52, the main species detected for both ChET and HeLaT are the C8 and C9 rings in the 700 to 900 kDa range. Finally, at 10-fold lower ChET and HeLaT protein concentration, the mass photometry distributions resolved dimers (1X), tetramers (2X), hexamers (3X), and the C8 (8X) and C9 (9X) rings, respectively (Fig. S8, *B* and *C*). We constructed binding isotherms to estimate K_d-app_ using the mass photometry data as explained in the Experimental procedures section (Fig. 6*D*). According to our analysis, on average, 70 to 80% of dimers are incorporated into rings at saturating Cp-52 concentrations, suggesting the presence of an inactive fraction of tubulin dimers that did not assemble into rings (∼20–30%). The weighted-mean mass isotherms in all three cases showed a sharp transition from free tubulin dimers to Cp-52 rings, characterized by a narrow range of Cp-52 concentrations near the midpoint of the isotherm. This and the low population of assembly intermediates observed in the probability density distributions suggested a cooperative behavior for the assembly reaction. Therefore, we analyzed the isotherm by nonlinear regression using a single binding-site model with cooperativity (Equation 1), and the resulting binding parameters are K_d-app-BBT_ = 172 ± 36 nM and *h*_BBT_ = 1.33 ± 0.34; K_d-app-ChET_ = 65 ± 17 nM and *h*_ChET_ = 1.45 ± 0.50; and K_d-app-HeLaT_ = 25 ± 7 nM and *h*_HeLaT_ = 1.34 ± 0.46 (Table 2).

**Table 2:** Apparent dissociation constant, cooperativity coefficients, and inactive fraction of Cp-52 binding

Parameter	BBT	ChET	HeLaT
K_d-app_^a^, nM	172 ± 36	65 ± 17	25 ± 7
Cooperativity coefficient (*h*)^a^^,^^b^	1.33 ± 0.34	1.45 ± 0.50	1.34 ± 0.46
Inactive fraction^c^	0.22	0.18	0.27

a The apparent dissociation constant K_d-app_ and the cooperativity coefficient were estimated by nonlinear regression and their uncertainties are the standard errors of the fits.

b The cooperativity coefficient (*h*) is used to improve the fitting of the experimental data, and it describes the steepness of the transition zone of the isotherm.

c The inactive fraction is the relative population of tubulin heterodimers that remained in solution and not forming rings at saturating Cp-52 concentrations (plateau region).

## Discussion

In this study, we report the first structure of blood cell-specific tubulin isotypes α1/β1 (orthologs of human genes TUBA1A/TUBB1) isolated from avian erythrocytes to a resolution of FSC_0.143_ = 3.2 Å, encompassing local resolutions from ∼2.5 to 4.5 Å. We also compared the complexes of Cp-52 with ChET to those formed with HeLaT. We will first discuss the structure of ChET before considering the interesting differences in the sizes of the ChET-Cp-52 and HeLaT-Cp-52 rings.

We took advantage of the ability of Cp-52 binding to induce the formation of rings that are amenable to single-particle cryo-EM due to their stability and abundance under our experimental conditions. The Cp-52 tubulin rings are also comparatively less polydisperse than other tubulin samples used for solving cryo-EM structures of tubulin since only two ring species are observed, having 8 or 9 αβ-tubulin heterodimers, which are easily separated using 2D classification. Moreover, given their smaller size than, for instance, Dolastatin-10 tubulin rings composed of 13 to 16 tubulin dimers, Cp-52 tubulin rings are more rigid and less prone to ring bending that can interfere with the single-particle workflow, reducing the resolution of the final reconstructions (39). MTs assembled *in vitro* are more heterogeneous, having 9 to 16 protofilaments or more (40). This new structure of ChET has a comparatively higher resolution than most previous cryo-EM structures of vitrified MTs, which generally range from 3.7 to 4.6 Å or lower (15, 18, 19), better resolution than Dolastatin-10 tubulin rings at 3.9 Å (41), and slightly better than the previous HeLaT-Cp-52 structure at 3.3 Å (21). Overall, it seems that Cp-52 tubulin rings can achieve the highest cryo-EM resolution of the curved tubulin protofilaments. Notably, recent cryo-EM structures of taxol-stabilized MTs bound to *Saccharomyces cerevisiae* kinesin-8 Kip3 (ScKip3, an MT minus end-binding protein) have reported resolutions in the range of 2.6 to 3.1 Å and revealed conformational changes in its motor core upon binding to tubulin (41). The potential interaction of MT binding proteins with Cp-52 tubulin rings remains to be elucidated, and may provide a platform for high-resolution studies of curved tubulin-MAPs interactions, much like the more gently curved Dolastatin-10 rings, which mimic the MT minus end and can bind the MT end-dependent ATPase of kinesin-13 motor protein (39).

For isotype structure/function modulation, ChET is informative to study because it is monoisotypic and contains blood-cell specific β1-tubulin, which is an ortholog to the most divergent β-tubulin (TUBB1) among the human tubulin isotypes (42). Previous *in vitro* studies have shown differences between ChET and tubulin from other sources. For example, ChET exhibits a very stable and almost dissociation-resistant αβ-tubulin heterodimer. In contrast, tubulins from other more heterogeneous sources such as the mammalian brain, chicken brain, HeLa cells, and from the protozoan *Leishmania tarentolae* readily dissociate into monomers under the same experimental conditions (43). Furthermore, ChET has binding and polymerization inhibition by colchicine-site drugs properties that are different from brain tubulin, showing selectivity towards antihelminthic benzimidazoles (44), which may be related to the residue substitution C239S found in the colchicine binding site of ChET compared to mammalian β-tubulins (45). These differences in dimer dissociation and colchicine-site drug binding have been attributed to the essentially different tubulin isotype composition used in these experiments.

The ChET α1/β1-tubulin heterodimer had conformational changes mainly located in the β1-tubulin subunit compared to HeLaT. This is consistent with the relatively large number of amino acid differences between the tubulin isotypes in ChET that have 2 and 53 substitutions in α1- and β1-tubulin, respectively, compared to the main tubulin isotypes in HeLaT. About half of the substitutions in ChET α1/β1-tubulin are surface exposed, while the rest are buried. Also, none of the sequence differences are near the ligands (GDP, GTP, and Cp-52). We observed several patches at the surface of ChET that are strikingly different in electrostatics and hydrophobicity potentials from the HeLaT structure. Analogous to what is observed in HeLaT-Cp-52 rings (21), and in dolastatin-10 αβ-tubulin rings (39), in ChET-Cp-52 rings, the interior and exterior surfaces of the ring correspond, respectively, to the MT outside and MT lumen (Fig. S6). Some amino acid substitutions are in these regions and might affect MT function. For instance, P32I and H83P are in the MT lumen surface loop and may affect loop conformation. F92H and S55Y are at the protofilament interface, which might be important for lateral interactions. K379I is located on the MT surface, which might be important to microtubule-associated protein (MAP) binding. Some sequence differences on the Cp-52 tubulin ring inner surface (MT outer surface) of the β subunit appear in the kinesin binding site (41), especially those on the C-terminal helix. Other MAPs that look like their binding sites might be affected are the motor dynein, the kinetochore component NDC80, doublecortin, and possibly others (46, 47). These sequence differences may relate to the functional and biological differences between HeLaT and ChET.

The structure of Cp-52 bound to ChET solved in this study revealed some differences compared with its conformation in the HeLaT-Cp-52 structure (PDB ID: 7LXB). However, the resolution of these cryo-EM reconstructions is limited and does not allow for a precise conformation of the drug to be resolved. Recently, a 2.2 Å X-ray structure of a cryptophycin-52 derivative (cryptophycin-52uD malate) bound to cow brain tubulin stabilized by stathmin and tubulin tyrosine ligase (T2R-TTL-cryptophycin complex) has been reported (48) and provides finer structural details but also reveals some differences. The T2R-TTL-cryptophycin complex contains two cryptophycin-52uD molecules bound to β-tubulin, and only one of them shares the same binding site observed in ChET-Cp-52 (this study) and in HeLaT-Cp-52 (21). Also, the T2R-TTL-cryptophycin complex is missing the α-tubulin binding site (48), while ChET-Cp-52 and HeLaT-Cp-52 show that both α- and β-tubulin are in contact with the Cp-52 drug. The other available structure for cryptophycin (cryptophycin-3) was solved by single-crystal X-ray crystallography in the unbound state (49). We compared the conformation of these cryptophycin structures using rigid body fitting to the ChET density map. We found that the cryptophycin conformation best explained by the cryo-EM density is the one bound to ChET solved in this study (Fig. S7). Moreover, the structural alignment of cryptophycin in the bound and unbound states revealed a significant conformational change of units A and B observed in the three available structures of bound cryptophycin-52 (Fig. S7), which are the same units where cryptophycin-52 showed a different orientation between ChET and HeLaT structures. This suggests conformational flexibility in the structure of tubulin-bound cryptophycin, specifically in units A and B. It has been previously observed that ligands can bind to proteins with ample conformational variability (36, 50). For example, ATP, NAD, and FAD, which are the most ubiquitous ligands in nature, are observed to bind with significant conformational variability to their receptors (50). Based on this previous work, our data support the scenario where the Cp-52 molecules can bind to different tubulins with slightly different conformations resulting from the changes in conformation from the unbound to bound states, as previously observed for other ligands. Furthermore, these findings suggest that the binding of the Cp-52 drug might serve to amplify the differential stabilities and conformational changes experienced by the different tubulin isotypes in response to environmental cues, similar to what is observed for taxol-stabilized MTs of tubulin isotypes α1/β4 and α1/β3 (20).

A side-by-side comparison between the cryo-EM structures of ChET- and HeLaT-Cp-52 rings composed of eight αβ-tubulin heterodimers showed a 5.9 Å smaller diameter for the former (measured at the subunit centers-of-mass; 5.2 Å at the POC). The intradimer and interdimer interfaces of ChET-Cp-52 rings are compacted compared to those of the HeLaT-Cp-52 rings, with greater differences found at the intradimer interface. This finding is similar to previous cryo-EM studies on the effect of tubulin isotype heterogeneity over MT structure, which observed differences at the interdimer interface on MTs composed of α1A/β3 compared to brain MTs (18), as well as interdimer interface compaction of MTs composed of different α-tubulin (19) or β-tubulin isotypes (15). The opposite was also observed: α1/β4 MT lattices are stabilized and expanded, instead of compacted, upon binding of taxol, while MT lattices composed of α1/β3 show stabilization but not expansion in the presence of stoichiometric amounts of the drug (20). The effect at the interdimer interface in the abovementioned studies differs from the observations we report here, where there is compaction at both intradimer and interdimer interfaces, and may reflect the difficulty of comparing the two structures with different geometry, that is, curved protofilaments in the Cp-52 tubulin rings *versus* straight protofilaments in the MTs.

The binding affinity of cryptophycin to tubulins other than brain tubulin is unknown. We used mass photometry to measure the Cp-52 apparent binding affinity to ChET and HeLaT (Fig. 6), by tracking their assembly into rings upon Cp-52 binding, observing small but consistent differences between them and compared to BBT. Mass photometry has been used to study the monomer-dimer equilibrium of bovine brain αβ-tubulin (51), and to characterize the oxidation-dependent assembly of dimeric *Arabidopsis thaliana* 2-cysteine peroxiredoxin into decameric rings (52) of similar shape as the tubulin Cp-52 rings. The Cp-52 binding affinity of BBT has been measured previously by three methods. Panda *et al.* (53) used radiolabeled [^3^H]cryptophycin-52 and Scatchard plot analysis to reveal a single high-affinity site and an association constant of 3.6 ± 1.0 × 10^6^ M^−1^ (278 ± 77 nM). Using the quenching of BBT intrinsic tryptophan fluorescence upon Cp-52 binding, the dissociation constant determined by the same group is 0.1 ± 0.01 μM (100 ± 10 nM) (53). Abel *et al.* (48) used isothermal titration calorimetry to measure the binding of cryptophycin to BBT, resulting in 97 ± 18 nM. Based on these data, one might expect a variation of the K_d_ in the range of ∼100 to 300 nM (or 2-3-fold), probably due to the system's complexity, which consists of various species, including the BBT heterodimer, the C8 and C9 rings, and other intermediate species. Here, we used mass photometry to track the coupled binding-assembly reaction of tubulin and Cp-52 for BBT, ChET, and HeLaT. In all three cases, we observed a single transition from 100% heterodimer in the absence of Cp-52, to a combination of 20 to 30% heterodimer and 70 to 80% rings at saturating Cp-52 concentrations. The fraction of tubulin dimers that do not assemble into rings, even at saturating Cp-52 concentrations, differs among the three tubulins analyzed in this study. Therefore, the isotherm saturation value (plateau) can differ for each tubulin depending on the fractional population of unassembled dimers. This suggests a more complex behavior of tubulin upon binding Cp-52 in solution than initially surmised, perhaps, indicating a population of inactive tubulin in our preparations, or it may reflect partial dissociation of the Cp-52 rings. Nevertheless, our mass photometry binding isotherm analysis showed that the BBT K_d-app_ measured in this study is essentially identical to those K_d_ values reported previously for the same protein. Interestingly, by fine-tuning the experimental conditions, using a low tubulin concentration and a nonsaturating Cp-52 concentration, at equilibrium, we could resolve intermediates of the ring assembly pathway that suggest a stepwise “Cp-52 binding and assembly” mechanism and confirm that the C8 and C9 rings are end products, given their abundance in these samples. To our knowledge, this is the first time such intermediates have been observed. Taken together, our mass photometry data indicate a small but detectable difference (2-3-fold) in K_d-app_ between HeLaT and ChET, and both have a lower K_d-app_ than BBT, namely a 5-fold and 3-fold difference, respectively. It is important to note that the K_d-app_ we measured here combines the processes of drug binding and protein assembly in a single parameter, and it may not reflect the intrinsic binding affinity of the Cp-52 to tubulin or reveal a biological difference among them. However, the amino acid arrangement of the Cp-52 binding site is nearly fully conserved among tubulin isotypes; therefore, a similar binding affinity is expected for these tubulins with different isotype compositions. Perhaps the differences observed are related to the subtle structural differences between the proteins and any PTMs near the binding site that can differentially modulate the ring assembly and the binding of the Cp-52 molecule.

The results presented here are significant because they demonstrate that the phenomenon of dimer spacing undergoing compaction (or expansion) of ∼2 Å does not require an MT lattice but can be observed by comparing two single protofilaments (HeLaT- *versus* ChET-Cp-52 rings) from tubulins with different isotype composition. We have chosen to refer to this as compaction of spacing in ChET-Cp-52 compared to the reference structure (HeLaT-Cp-52), but the essential point is the difference in the sizes of the two rings composed of eight tubulin heterodimers each. Our analysis indicates that the main differences between the ChET and HeLaT dimers are in the β-tubulin amino acid sequences that result in conformational changes of the subunit that differ between both tubulins. This new structure of β1-tubulin (encoded by TUBB1), which is the predominant isotype in the marginal band of platelets, will provide insights into β1-tubulin role in diseases such as platelet anisocytosis and congenital thrombocytopenia produced by mutations in TUBB1 (10, 11) but also its potential role in chemotherapy-induced thrombocytopenia in patients under cancer treatment (54). Our results also highlight the benefits of using naturally occurring monoisotypic tubulin (in the case of ChET) to reduce structural heterogeneity, and of using tubulin-specific drugs such as cryptophycin for inducing structures well-suited for single-particle cryo-EM analysis. This has allowed us to detect a previously undescribed phenomenon of compaction/expansion of tubulin interfaces in a single protofilament (though curved), that is structurally related to what has been observed for straight MTs, and opens new routes to more deeply understand the structure-function relationships of the various tubulin isotypes found *in vivo*.

## Experimental procedures

### Preparation of blood cell-specific tubulin and formation of cryptophycin-52 rings

Blood cell-specific α1/β1−tubulin was purified from ChET (#33131-1, Pel-Freez Biologicals), as described previously (43, 55). Purified ChET was stored in PM buffer (0.1 M Pipes-KOH, pH 7.0, 1 mM MgCl_2_) at −80 °C. Protein concentration was estimated using the Bradford assay (Bio-Rad) with bovine serum albumin as the calibration standard (#23209, Thermo Fisher Scientific). Cp-52 was dissolved in dimethyl sulfoxide (DMSO) at 1 mM and stored at −20 °C. For preparing ChET-Cp-52 rings, the purified protein was centrifuged at 100,000*g* for 20 min at 4 °C in a benchtop Optima MAX ultracentrifuge (Beckman Coulter). The supernatant was transferred to a new tube. ChET was diluted in PM buffer to 5 μM and Cp-52 was added at a final concentration of 10 μM, directly from the 1 mM stock in DMSO. The final DMSO concentration in the reaction mixture was 1%. The mixture was incubated for 30 min at 25 °C before downstream experiments.

### Negative stain electron microscopy

Ten microliters of the ChET Cp-52 mixture were deposited on glow-discharged copper grids (carbon film, 400-mesh, Electron Microscopy Sciences) and incubated for 30 s. The grid was washed twice with water and then stained with uranyl acetate 2% in water (Electron Microscopy Sciences) for 30 s. The grid was dried by blotting against filter paper (#1 Whatman). Grids were observed in an FEI Tecnai T12 microscope at 120 kV equipped with an Ultrascan camera 2k × 2k (Gatan). Images were acquired at a nominal magnification of 42k. Images were collected using an exposure time of ∼1 s and drift corrected using Digital Micrograph software (Gatan; https://www.gatan.com/products/tem-analysis/gatan-microscopy-suite-software).

### Sample vitrification and cryogenic electron microscopy

Starting from the same mixtures used for negative stain EM, ∼3.5 μl of the sample were deposited onto Quantifoil R 1.2/1.3400-mesh copper grids + 2 nm ultrathin carbon film (Electron Microscopy Sciences), previously hydrophilized by a 15 s glow discharge cycle (15 mA) in 0.02 mbar air vacuum (easiGlow, PELCO). The loaded grids were blotted from both sides with filter paper (#1, Whatman) and vitrified in liquid ethane using a Vitrobot Mark IV plunge freezer (Thermo Fisher Scientific) with the chamber environment set at 95% humidity and at 4 °C. Immediately after, the grids were clipped and stored in the liquid nitrogen gas phase until use. Data collection was done at the University of Virginia Molecular Electron Microscopy Core facility, using an FEI Titan Krios 300 kV microscope equipped with a K3 direct electron detector (Gatan) configured in electron counting mode and using 10 eV slit energy filter. Subsequently, 7842 movies were collected at 40 frames using an exposure time of 2.88 s with a total dose of 50 e^−^/Å^2^. The target defocus varied in the range −1.8 to −0.8 μm. The nominal magnification used was 1,050,00× with a calibrated pixel size of 0.83 Å/pixel.

### Cryo-EM image processing

All the steps of single particle analysis, including movie alignment, contrast transfer function (CTF) correction, particle picking, particle extraction, 2D classification, *ab initio* reconstruction, and 3D volume classification and refinement, were performed with CryoSPARC v.4.3.1 (https://cryosparc.com/updates) (29), hosted by the NIH HPC Biowulf cluster. Movie alignment and CTF correction steps were made using the patch motion correction and patch CTF correction algorithms, respectively. Particle picking was done in two steps, first by manually selecting ∼2773 particles consisting of top and side views of vitrified ChET-Cp-52 rings, followed by 2D classification. A subset of eight classes was chosen to build a template, which was used in the second step of automated templated-based particle picking, resulting in ∼1.8 million picked particles that were extracted using a box of 548 × 548 Å. Multiple cycles of 2D classification selected subsets of particles with 8-fold and 9-fold symmetry, consisting of 168,140 particles and 181,286 particles, respectively. These particle stacks were used for 3D *ab initio* reconstruction, followed by 3D classification into three classes. The class with the highest homogeneous refinement resolution was selected in both cases, comprising 70,192 and 64,200 particles with 8-fold and 9-fold symmetry, respectively. The final 3D refinement step used the nonuniform refinement job, imposing C8 and C9 symmetry, respectively. The resulting 3D reconstructions have resolution FSC _0.143_ = 3.20 Å for the C8-ring and FSC _0.143_ = 3.54 Å for the C9-ring (see Table S1 for details). The local resolution maps were obtained using CryoSPARC’s “local resolution estimation” job. The final sharpened maps were obtained from the half-maps of the refined 3D reconstructions with RELION’s postprocessing tools (https://relion.readthedocs.io/en/release-4.0/Installation.html) (56). The absolute voxel sizes of the reconstructed cryo-EM maps of ChET-Cp-52 (EMD-45263) and HeLaT-Cp-52 (EMD-53569) were determined independently with the high-resolution structure method as shown in Fig. S9 (57, 58).

### Atomic model building and refinement

An initial homology model of α1/β1-tubulin was built using the SWISS-MODEL workspace (https://swissmodel.expasy.org/interactive) (59) with the *Gallus gallus* amino acid sequences of α1-tubulin isotype (tubulin alpha-1A chain, gene TUBA1A, accession NP_001292201.2, UniProt P02552) and of β1-tubulin isotype (or tubulin beta-6 chain, or class VI, gene TUBB1, accession NP_990776.1, UniProt P09207). These genes from *Gallus gallus* are orthologs of human genes TUBA1A and TUBB1. The structure of the Cp-52 molecule was obtained from the RCSB PDB database (60) as residue YGY extracted from PDB ID: 7LXB (21). The starting ChET α1/β1-tubulin dimer was built by aligning the models of both α- and β-tubulins to the asymmetric unit of 7LXB to generate the asymmetric unit of ChET including α1-and β1-tubulins, the ligands GDP, GTP, and Cp-52 (residue YGY). Subsequently, the ChET dimer Cp-52 homology model was fitted to the C8 postprocessed cryo-EM density map in UCSF ChimeraX v1.5 (https://www.cgl.ucsf.edu/chimerax/download.html) (61). This model was then further aligned to the C8 map using PHENIX (https://phenix-online.org/download) (62), and the map was cropped to obtain the cryo-EM density corresponding to the asymmetric unit alone. Several cycles of real-space refinement in PHENIX, followed by model building in Coot (https://www2.mrc-lmb.cam.ac.uk/personal/pemsley/coot/) (63) were performed to get the final refined atomic model of the ChET-Cp-52 asymmetric unit. Model building into the full C8 ChET-Cp-52 ring cryo-EM map was started by rigid-body fitting eight copies of the final refined model of the ChET-Cp-52 asymmetric unit, followed by real-space refinement in PHENIX and model building in Coot. An equivalent approach was used to build the C9 ChET-Cp-52 ring atomic model. The real-space refinement in PHENIX and model building in Coot included the ligands GDP, GTP, and Cp-52 (YGY) in all the refinement steps. Model quality was judged using PHENIX’s MolProbity report and with the wwPDB validation server (https://validate-rcsb-1.wwpdb.org/).

### Mass photometry measurements and data analysis

BBT was purchased from PurSolutions, LLC; and tubulin from HeLaT was purified as previously described from cultured cells (64). Tubulin in storage buffer was exchanged into fresh and filtered PM buffer (0.1 M Pipes-KOH, pH 6.9; 1 mM MgCl_2_) using a spin column (Zeba, 100k molecular weight cut-off) and then centrifuged at 100k*g* in a benchtop Optima MAX ultracentrifuge (Beckman Coulter) for 20 min at 4 °C. The supernatant was transferred to a new tube, and the tubulin concentration was estimated using absorbance (Extinction coefficient 280 nm = 110,000 M^−1^ cm^−1^). Tubulin was diluted to the target working concentration (1 μM for binding isotherms and 100 nM for detecting intermediates) in PM buffer and incubated with varying concentrations of Cp-52 in the range 0 to 10 μM at room temperature for 30 to 45 min prior to loading into the instrument. Cp-52 was added directly from a concentrated stock in DMSO and the final DMSO concentration did not exceed 2% in the mixtures. After this incubation, the samples were further diluted to 50 nM protein to be within the quantitation range of the instrument, and the data were integrated for 60 s for each acquisition using the OneMP mass photometer (Refeyn). Raw contrast values were converted to raw molecular masses using a standard mass calibration curve previously collected under the same conditions. The raw mass photometry histograms were exported using Discover MP (Refeyn; https://www.refeyn.com/about-mass-photometry) and then plotted and analyzed using the Seaborn statistical data visualization library in Python (65). The raw mass histograms were fitted with the kernel density estimate and then normalized to a total area of one to generate the probability density distributions. The distributions were multiplied by the number of tubulin heterodimers they contain (raw mass divided by 100 kDa) and then re-plotted to obtain the weighted-mass probability density distributions, with each species amplitude (equivalent to histogram counts) representing the total number of tubulin heterodimers contained instead of the total counts of the species. The weighted mean mass of each condition was obtained from the weighted-mass probability density distributions and plotted as a function of Cp-52 concentration to obtain the weighted-mean mass isotherms. The fractional incorporation isotherm was calculated by dividing the weighted-mean masses by that of the ring species at saturating Cp-52 concentrations (3–10 μM). The isotherms were fitted using a single binding-site model with a cooperativity coefficient, and the 50% value of the isotherm represented the K_d-app_ of Cp-52 binding (Equation 1) (66):

(1) $$F_{b}=F_{0}+\left[\frac{F_{b-\max}\times {\left[ligand\right]}^{h}}{{\left(K_{d-app}\right)}^{h}+{\;\left[ligand\right]}^{h}}\right]$$

With F_b_, the fraction incorporated into rings; F_0_, the fraction incorporated in the absence of Cp-52; F_b-max_, the fraction incorporated at saturating Cp-52 concentration; K_d-app_, the concentration of Cp-52 that produces a 50% saturation; [ligand], the concentration of Cp-52 in nM units, and h, is the cooperativity coefficient that serves the purpose of improving the fits to the experimental data and describes the steepness of the transition region (66).

## Data availability

Maps have been deposited in the EMDB, https://www.ebi.ac.uk/pdbe/emdb/ (accession nos. EMD-45263 and EMD-45265). Models have been deposited in the PDB, https://www.ebi.ac.uk/pdbe/ (PDB ID codes: 9C6R, ChET-Cp-52 C8 ring; 9C6S, ChET-Cp-52 C9 ring).

## Supporting information

This article contains supporting information.

## Conflict of interest

The authors declare that they have no conflicts of interest with the contents of this article.

## Supporting information

Supplemental Figs. S1−S9 and Table S1
