# Supplementary material for: Structure of blood cell-specific tubulin and demonstration of dimer spacing compaction in a single protofilament
Source: J Biol Chem. 2024 Dec 24;301(2):108132. doi: 10.1016/j.jbc.2024.108132 (PMC11791314; doi:10.1016/j.jbc.2024.108132)
Supplement: Supplemental Figs. S1−S9 and Table S1 [file mmc1.docx]

**Supporting Information**

**Title:** Structure of blood cell-specific tubulin and demonstration of dimer spacing compaction in a single protofilament

**Authors:** Felipe Montecinos1, Elif Eren1, Norman R. Watts1, Dan L. Sackett2 and Paul T. Wingfield1

**Running title:** Blood cell tubulin structure bound to Cryptophycin-52

**Affiliations:**

1Protein Expression Laboratory, National Institute of Arthritis and Musculoskeletal and Skin Diseases, National Institutes of Health, Bethesda, Maryland, USA

2Division of Basic and Translational Biophysics, Eunice Kennedy Shriver National Institute of Child Health and Human Development, National Institutes of Health, Bethesda, Maryland, USA

**Supplementary Table 1.** Cryo-EM data collection, processing, refinement, and validation statistics.

**Supplementary Figure 1**. Cryo-EM map and atomic structure of ChET α1/β1-tubulin structure refined with C9 symmetry.

**Supplementary Figure 2**. ChET-Cp-52 structural flexibility and amino acid conservation among tubulin isotypes.

**Supplementary Figure 3**. Sequence alignment of αβ−tubulin isotypes found in ChET, HeLaT, and tubulin from brain tissue.

**Supplementary Figure 4**. Determination of the dimer spacing in Cp-52 tubulin rings and comparison to GDP-microtubules.

**Supplementary Figure 5**. Conformational changes in the ChET-Cp-52 α1/β1−tubulin heterodimer compared to HeLaT-Cp-52

**Supplementary Figure 6**. Side-by-side comparison of the calculated electrostatic and hydrophobicity potentials for HeLaT-Cp-52 and ChET-Cp-52 tetrameric structures.

**Supplementary Figure 7**. Comparison between conformations of various cryptophycins and fit-in-map cross-correlation to the Cryptophycin-52 cryo-EM density in ChET.

**Supplementary Figure 8**. Mass photometry kernel density distributions of tubulin bound to Cp-52 at non-saturating protein concentrations.

**Supplementary Figure 9.** Determination of the absolute voxel scale of ChET-Cp-52 and HeLaT-Cp-52 refined cryo-EM maps.

**Supplementary Tables**

| **Table S1**. Cryo-EM data collection, processing, refinement, and validation statistics | | |
| --- | --- | --- |
| Deposition Id | C8-ring  EMD-45263  PDB: 9C6R | C9-ring  EMD-45265  PDB: 9C6S |
| Data collection |  |  |
| Magnification | 105,000 | 105,000 |
| Voltage (kV) | 300 | 300 |
| Number of frames | 40 | 40 |
| Total dose (e−/Å^2^) | 50 | 50 |
| Dose per frame (e−/Å^2^) | 1.25 | 1.25 |
| Exposure time (s) | 2.88 | 2.88 |
| Defocus range (mm) | -1.8 to -0.8 | -1.8 to -0.8 |
| Pixel size (Å) | 0.83 | 0.83 |
| Data processing |  |  |
| Symmetry | C8 | C9 |
| Initial particle number | 168,140 | 181,286 |
| Final particle number | 70,192 | 64,200 |
| Resolution (Å) | 3.20 | 3.54 |
| Fourier shell correlation threshold | 0.143 | 0.143 |
| Refinement |  |  |
| Map sharpening *b*-factor (Å^2^) | -83.2 | -106.9 |
| Model composition |  |  |
| Nonhydrogen atoms | 55,352 | 62,271 |
| Protein residues | 6,944 | 7,812 |
| Nucleotide (GTP/GDP) | 8/8 | 9/9 |
| Ligand | 8 | 9 |
| B-factors (Å^2^) |  |  |
| Protein | 79.44 | 112.95 |
| Nucleotide (GTP/GDP) | 49.14/44.62 | 88.82/75.46 |
| Ligands | 53.00 | 80.59 |
| RMSD |  |  |
| Bond length (Å) | 0.005 | 0.003 |
| Bond angles (°) | 0.816 | 0.715 |
| Validation |  |  |
| Model-to-map fit CC protein | 0.82 | 0.65 |
| Model-to-map fit CC nucleotide (GTP/GDP) | 0.81/0.77 | 0.62/0.61 |
| Model-to-map fit CC ligand | 0.79 | 0.64 |
| Molprobity score | 1.31 | 1.36 |
| Clashscore | 5.67 | 6.58 |
| Side-chain outliers (%) | 0.20 | 0.0 |
| Ramachandran plot |  |  |
| Favored (%) | 98.35 | 98.79 |
| Allowed (%) | 1.62 | 1.17 |
| Disallowed (%) | 0.03 | 0.04 |


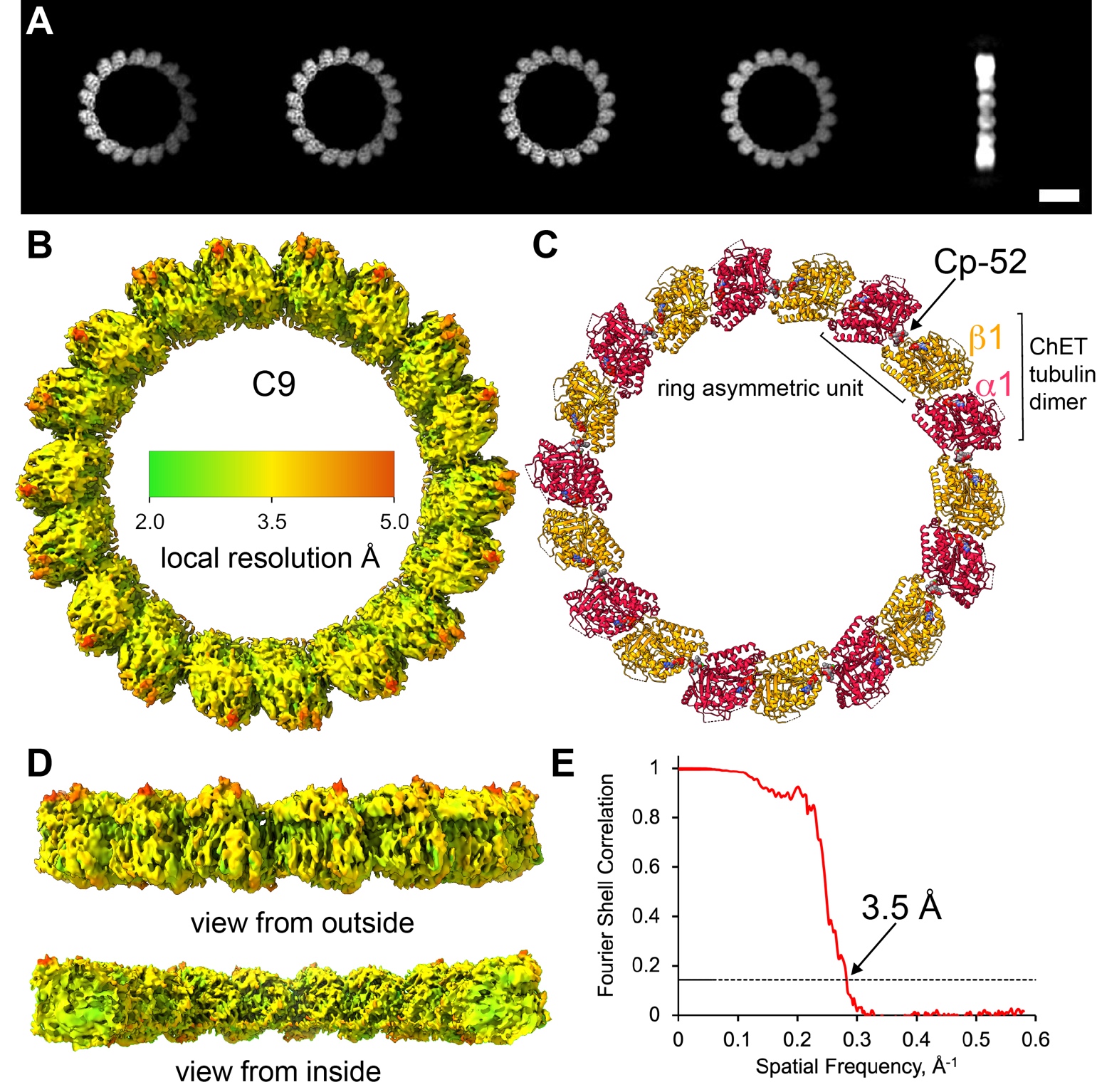


**Supplementary Figure 1**. Cryo-EM map and atomic structure of ChET α1/β1-tubulin structure refined with C9 symmetry. **A**, selected 2D classes obtained from the single-particle analysis cryo-EM workflow in CryoSPARC v4.3.1. Tubulin rings with C9 symmetry (i.e., nine ChET dimers) are shown. **B**, sharpened cryo-EM map of C9 ChET-Cp-52, colored according to local resolution estimation. **C**, refined atomic structure of C9 ChET-Cp-52 showing α1-tubulin (red) and β1-tubulin (orange) in ribbon representation, and Cp-52, GDP, and GTP in VDW representation, respectively. **D**, views of the C9 ChET-Cp-52 cryo-EM map from outside and inside of the ring, colored by local resolution estimation. **E**, Fourier shell correlation curve for estimating the cryo-EM map resolution, resulting in an FSC _0.143_ = 3.5 Å.


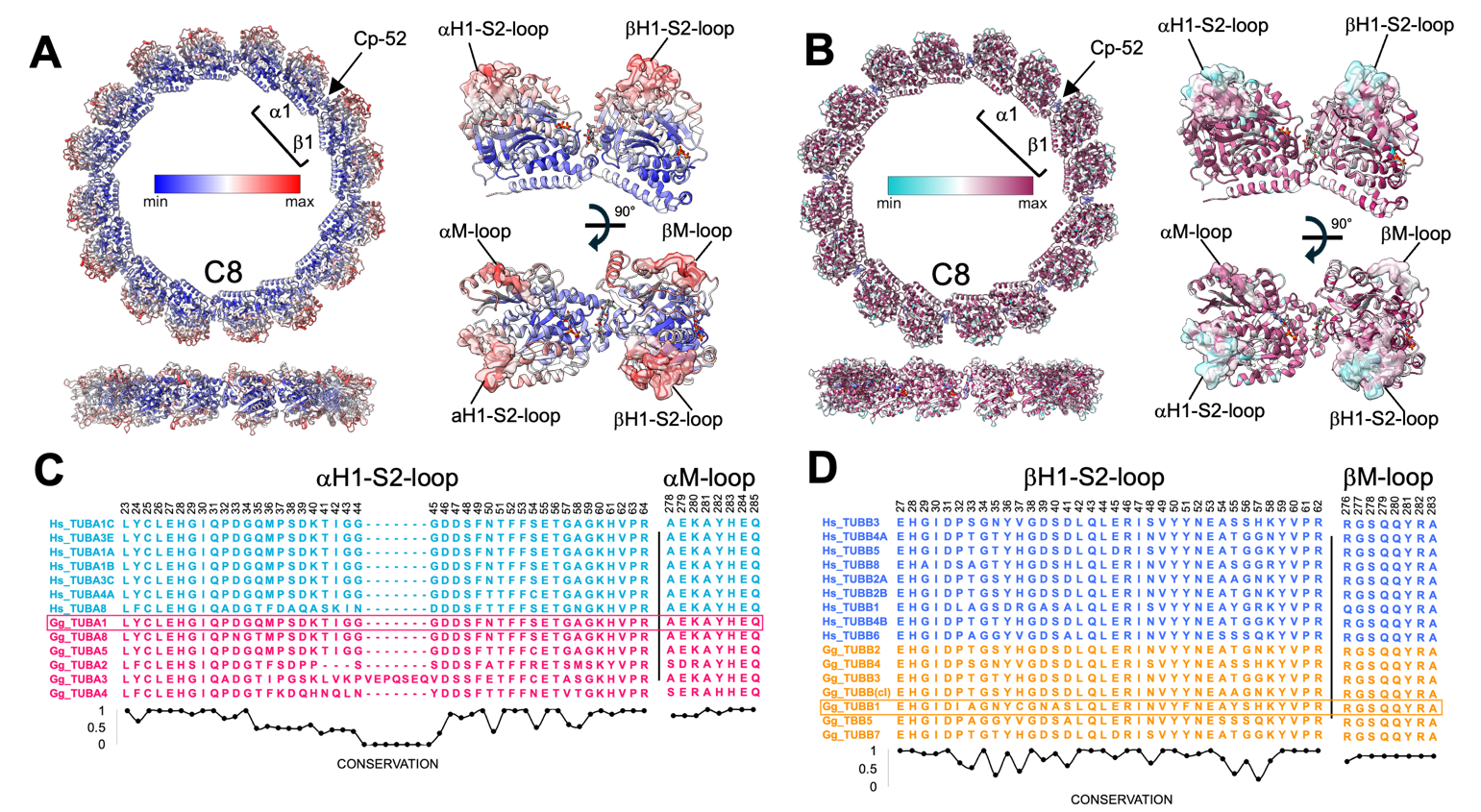


**Supplemental Figure 2**. ChET-Cp-52 structural flexibility and amino acid conservation among tubulin isotypes. **A,** the atomic structure of ChET is colored according to the refinement B-factor. The areas of high structural flexibility (high B-factor) in ChET-Cp-52 (the asymmetric unit is shown to the right) are localized at regions containing generally unstructured connecting loops, like the loop connecting strand S1 and helix H2 (H1-S2-loop) and the M-loop. **B**, the atomic structure of ChET-Cp-52 is colored according to sequence conservation between α/β isotype subfamilies from *Homo sapiens* and *Gallus gallus*. While the M-loop sequence shows high sequence conservation among tubulin isotypes, the unstructured H1-S2-loop displays a lower overall conservation. **C** and **D**, amino acid sequence alignment H1-S2-loop and M-loops. The conservation index was calculated as the reciprocal of the normalized Shannon entropy (using 20 as the number of amino acid types).

**
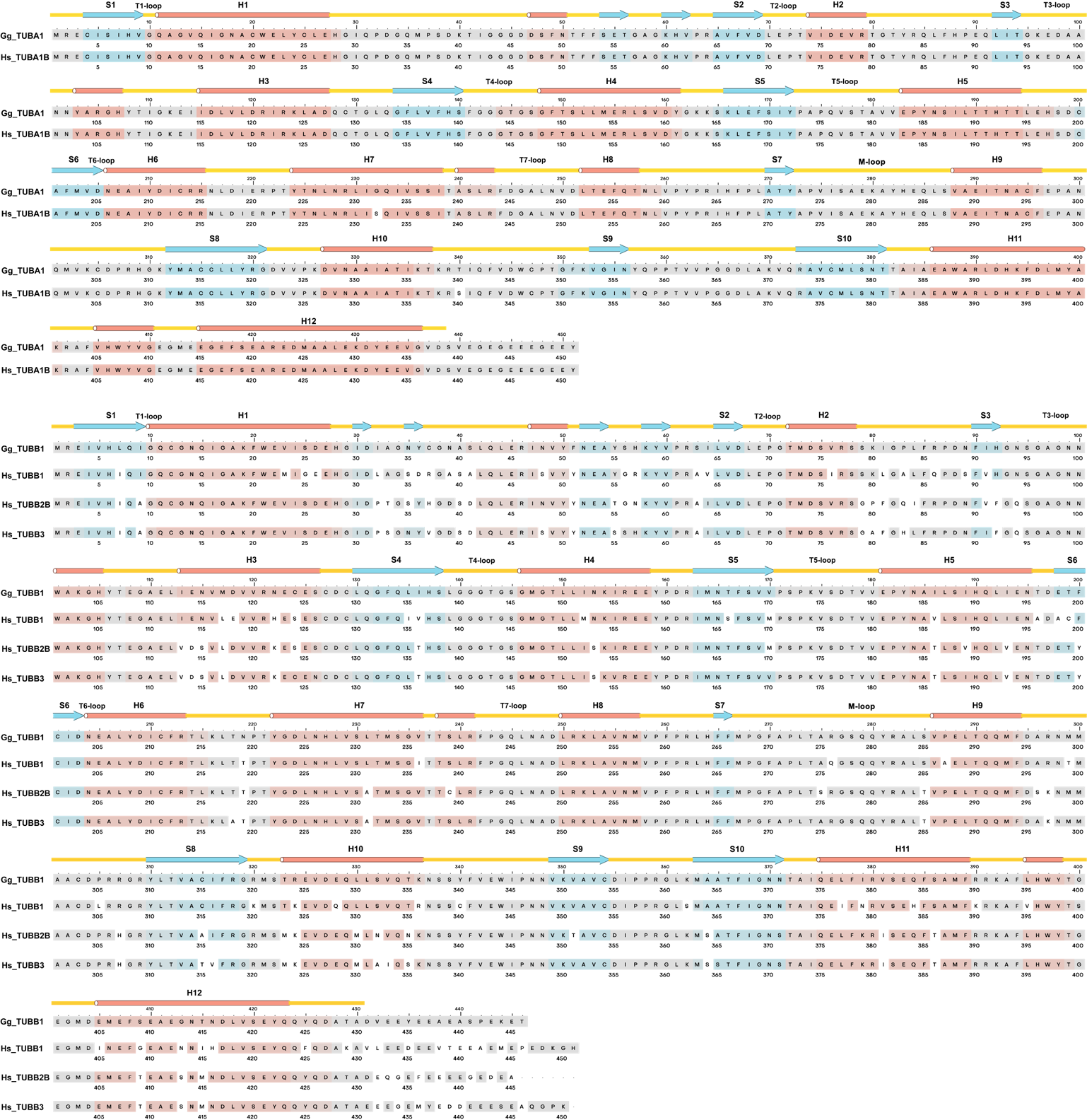
**

**Supplementary Figure 3**. Sequence alignment of αβ−tubulin isotypes found in ChET, HeLaT, and tubulin from brain tissue. The secondary structure assignment corresponding to ChET α1/β1−tubulin are shown. Helices, strands, and loops (unstructured) are colored red, blue, and yellow, respectively. The amino acids with a white background are different than those in ChET (*Gallus gallus*, Gg_TUBA1/Gg_TUBB1). Tubulins from humans are Hs_TUBB1, Hs_TUBB2B, and Hs_TUBB3. The sequence alignment with secondary structure assignments was prepared with BIOVIA Discovery Studio Visualizer 2021.


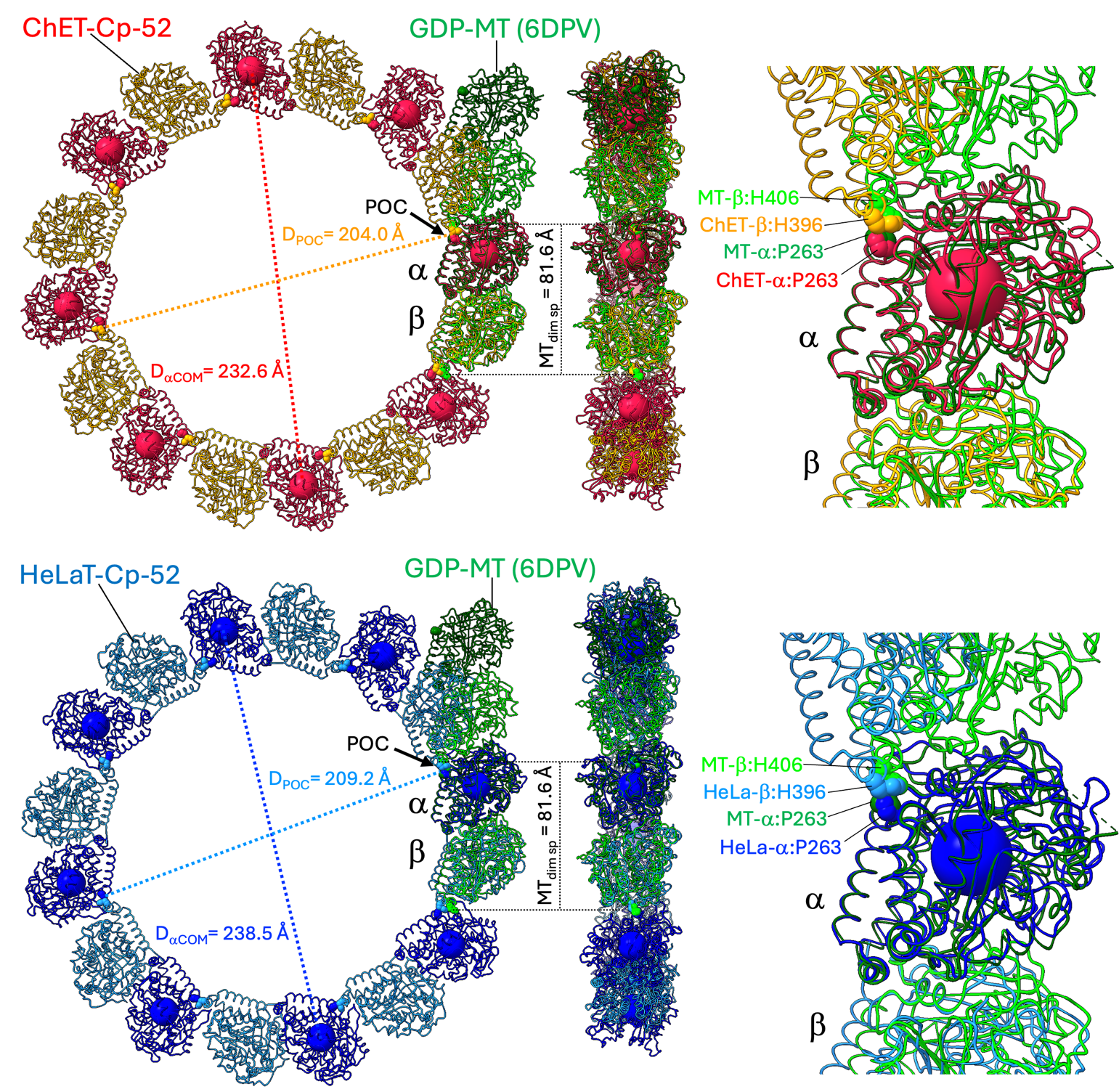


**Supplementary Figure 4**. Determination of the dimer spacing in Cp-52 tubulin rings and comparison to GDP-microtubules. The structures of Cp-52 rings for ChET (upper panel) and HeLaT (lower panel) were aligned to the structure of the GDP-MT (6DPV) through their α−tubulin subunits. The center-of-mass for each α−tubulin subunit in the Cp-52 tubulin ring is represented by the red (ChET) and blue (HeLaT) spheres. The point-of-contact (POC) at the longitudinal (interdimer) interface is defined by the center-of-mass of the pair of residues in the Cp-52 tubulin ring (VDW representation) that align closest to those in the GDP-MT structure, and these residues are located on the internal side of the ring, corresponding to the MT outside. A zoomed-in view of the POC residues is shown on the right side of each panel. For ChET and HeLaT the POC residues are α:P263 and β:H396, and for GDP-MT, the residues are α:P263 and β:H406, which are also shown in VDW representation. The Cp-52 tubulin ring diameter measured using the α−tubulin centers-of-mass (D_αCOM_) is greater than when measured using the POC residues (D_POC_) because the latter are located further inside the ring. The GDP-MT dimer spacing measured using the centers-or-mass of the POC residues is 81.6 Å. The Cp-52 tubulin ring dimer spacing is calculated from the circumference defined by the D_POC_ and using the formula Dπ/8, yielding 82.2 Å dimer spacing for HeLaT-Cp-52, and 80.1 Å for ChET-Cp-52.


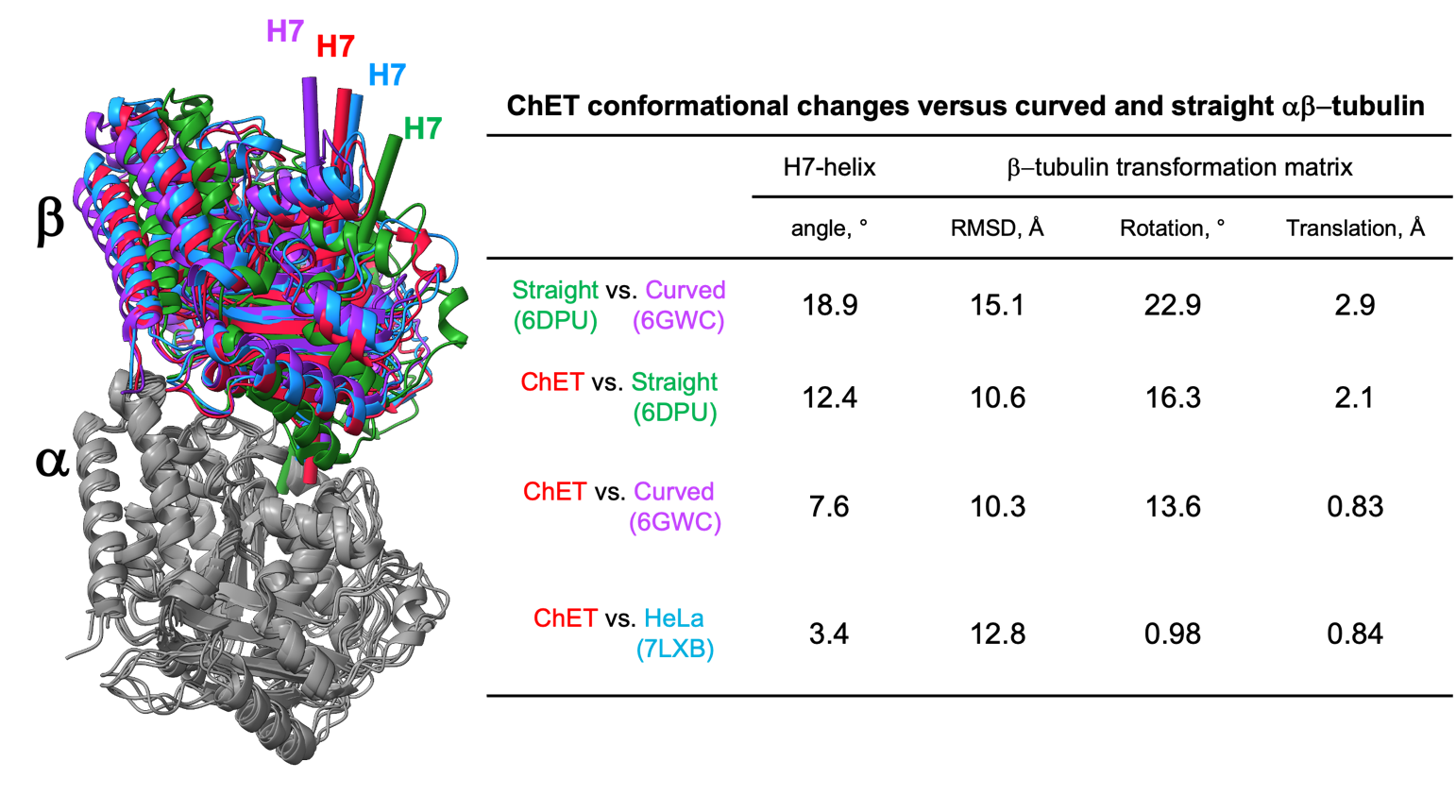


**Supplementary Figure 5.** Conformational changes in the ChET-Cp-52 α1/β1−tubulin heterodimer compared to HeLaT-Cp-52 (PDB ID: 7LXB, blue), straight αβ−tubulin (6DPU, green), and curved αβ−tubulin (6GWC, purple). Tubulin dimers in the curved and straight states were structurally aligned with ChET-Cp-52 (red) using the α−tubulin chain as reference (α−tubulin in gray). The secondary structure element helix-H7, which is considered the boundary between the two main N- and C-terminal domains of tubulin, was used as a geometric axis to calculate the orientation between the α−tubulin and β−tubulin subunits in ChimeraX v1.8. The angles between the H7 axes (depicted as cylinders) and the transformation matrices required to match the positions and orientations of the β−tubulin subunits after aligning the four tubulin dimers at their α−subunits, are given in the table.


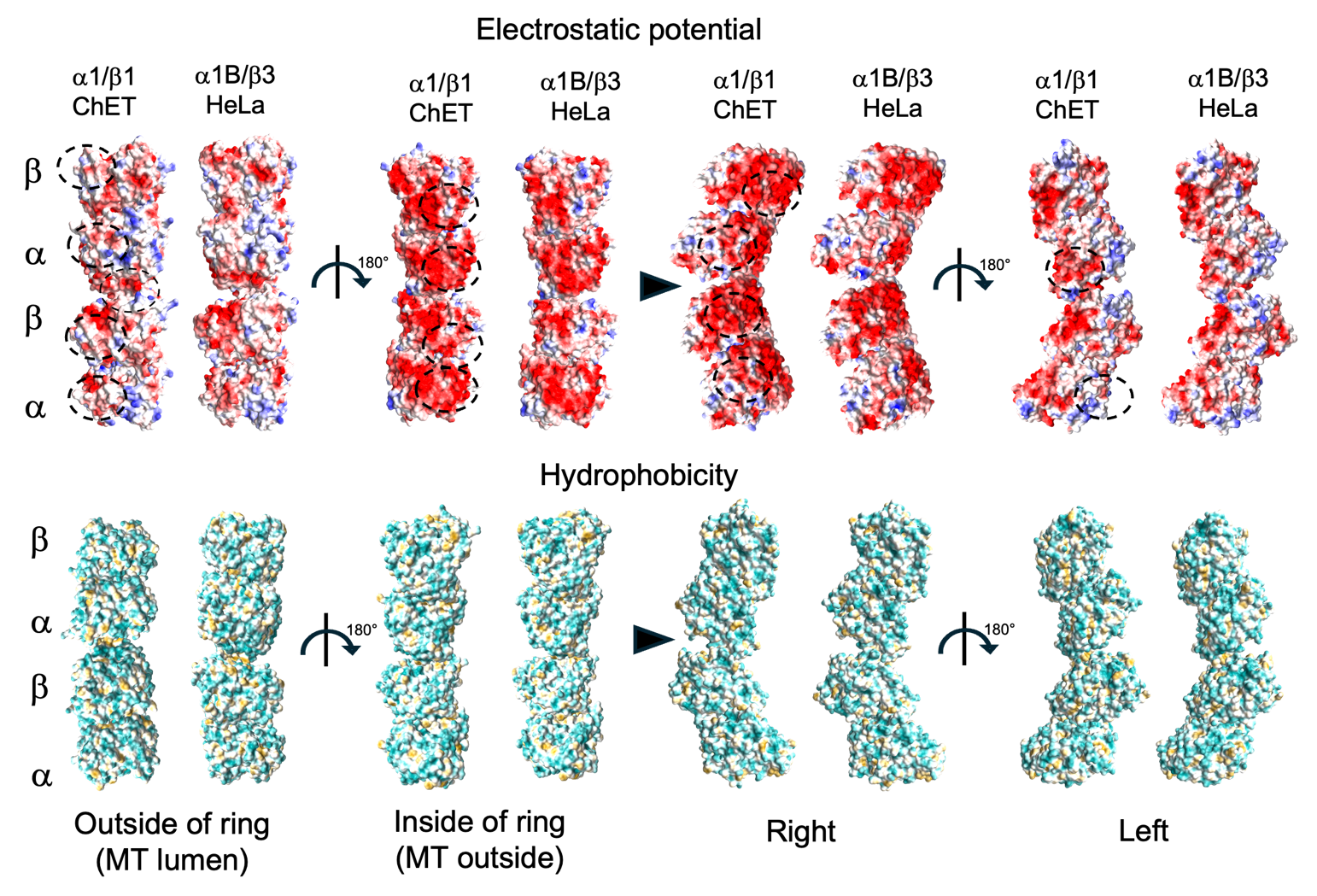


**Supplementary Figure 6**. Side-by-side comparison of the calculated electrostatic (top) and hydrophobicity (bottom) potentials for ChET-Cp-52 and HeLaT-Cp-52 tetrameric structures. A dashed oval marks patches that are visibly different between both structures. The Cp-52 binding site is in between the two tubulin dimers (arrowhead).


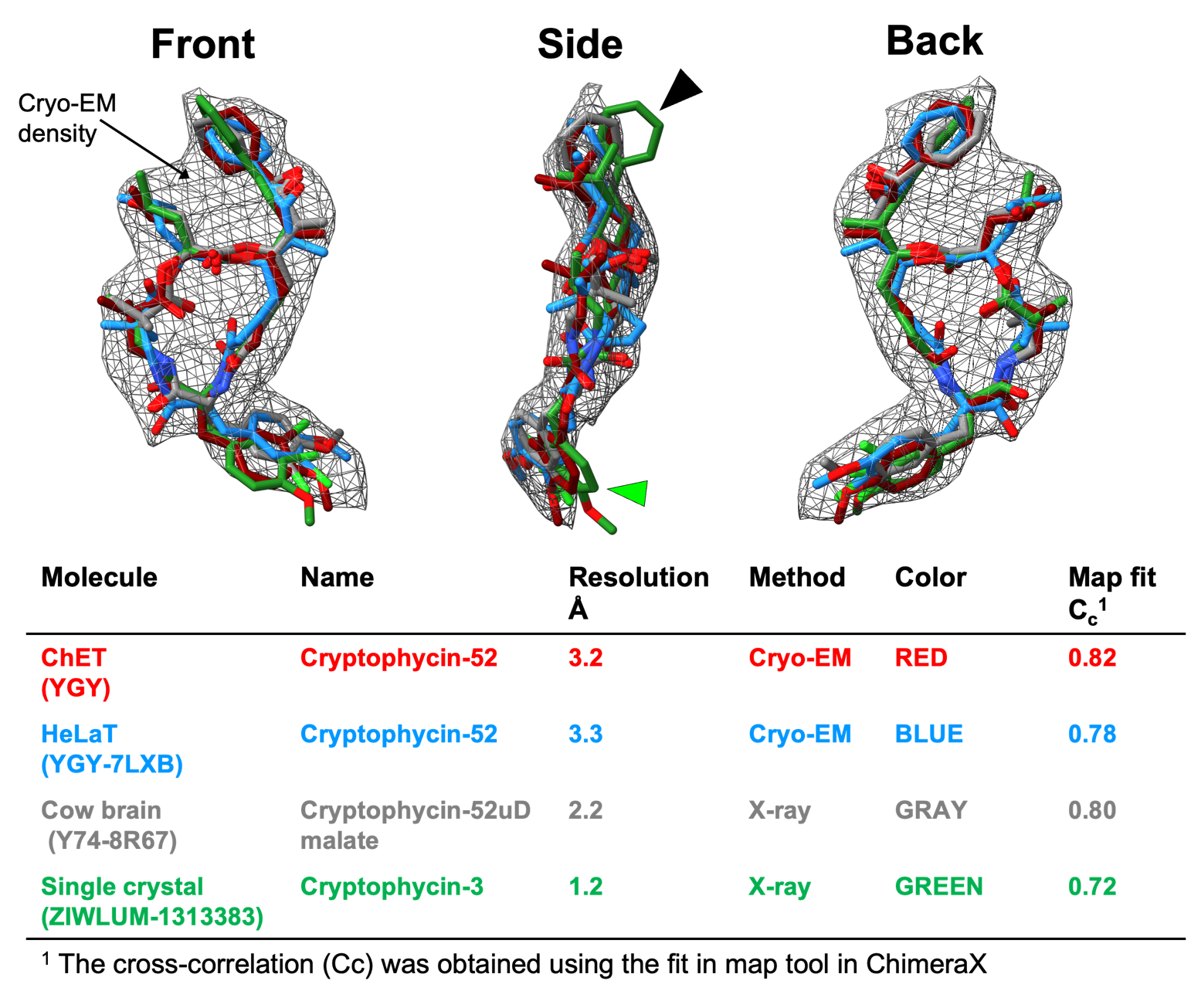


**Supplementary Figure 7**. Comparison between conformations of various cryptophycins and fit-in-map cross-correlation to the Cryptophycin-52 cryo-EM density in ChET. The black and green arrowheads point to units A and B of the cryptophycin molecule that experience a significant conformational change between the unbound (green structure) and bound states (red, blue and gray structures). The Cryptophycin-52 structures were obtained from this study (red) and from PDB ID: 7LXB (blue). The structure of Cryptophycin-52uD malate was obtained from PDB ID: 8R67 (bound to cow brain tubulin, gray). The structure of Cryptophycin-3 was obtained from entry ZIWLUM (1313383, green) in the Cambridge Crystallographic Data Centre (CCDC).

**Supplementary Figure 8.** Mass photometry kernel density distributions of tubulin bound to Cp-52 at non-saturating protein concentrations of BBT (**A**), ChET (**B**), and HeLaT (**C**). The concentration of tubulin used in these experiments is 100 nM, and that of Cp-52 is 100 nM, in PM buffer at room temperature. The mass photometry measurements revealed the presence of intermediates of the assembly reaction driven by Cp-52 binding. According to the calibration, the peaks correspond to single tubulin heterodimers (1X), dimers of heterodimers (2X), and so on, and C8-rings (8X) and C9-rings (9X).


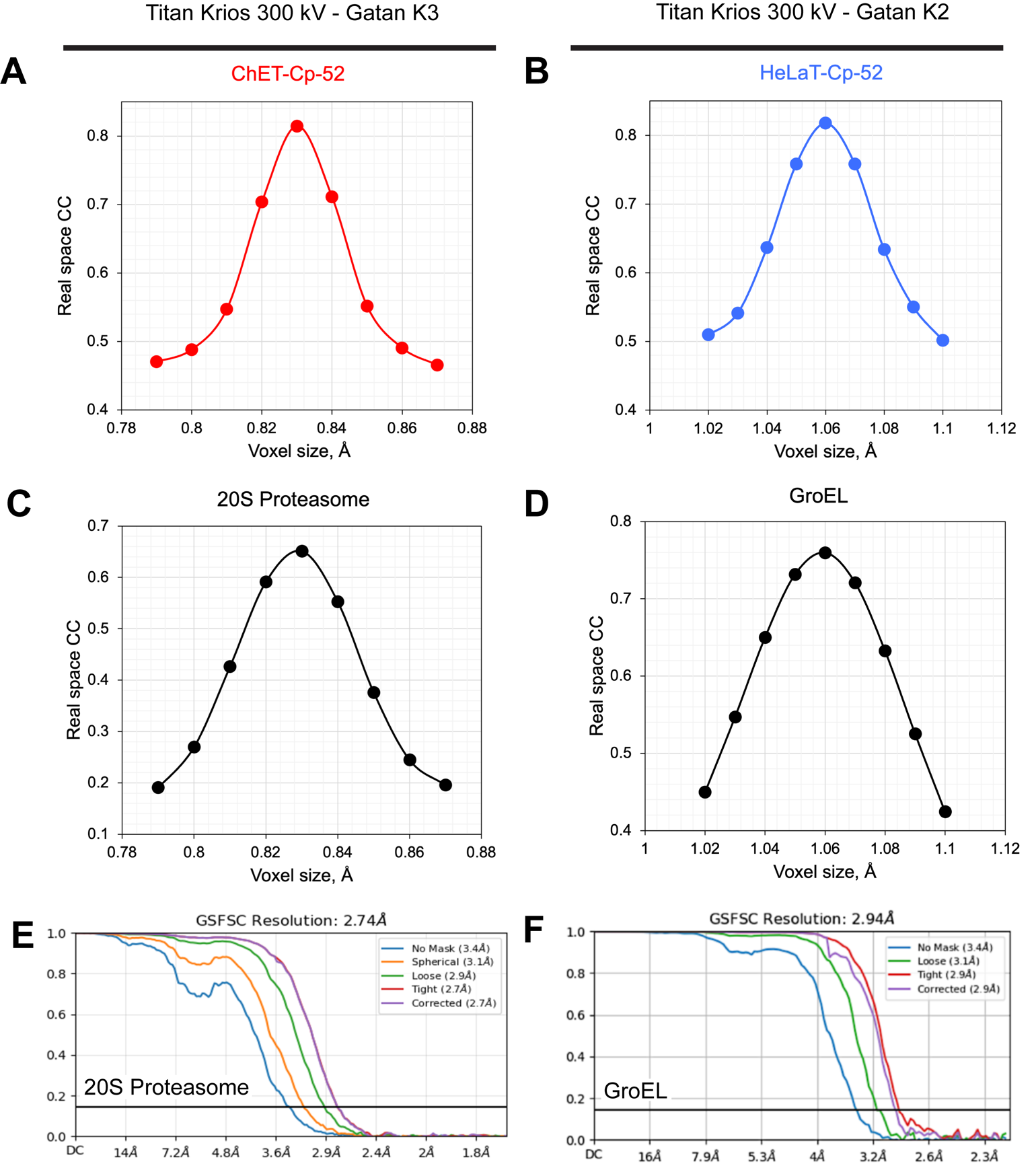


**Supplementary Figure 9.** Determination of the absolute voxel size of ChET-Cp-52 and HeLaT-Cp-52 refined cryo-EM maps. The real space cross-correlation (CC) between the voxel-rescaled maps and the atomic model-calculated maps was obtained with the software Chimera v1.11. **A**, the CC between the voxel-rescaled EMD-45263 cryo-EM map and the PDB 9C6R model-calculated map (ChET-Cp-52), resulting in an optimal voxel size of 0.83 Å. **B**, the CC between the voxel-rescaled EMD-23569 cryo-EM map and the PDB 7LXB model-calculated map (HeLaT-Cp-52), showing the optimal voxel size of 1.06 Å. **C**, the CC between the voxel-rescaled 2.74 Å 20S Proteosome refined cryo-EM map (unpublished) and the 2.14 Å 20S Proteosome X-ray crystal structure (PDB ID: 8BZL) model-calculated map, showing an optimal voxel size of 0.83 Å. **D**, the CC between the voxel-rescaled 2.94 Å GroEL refined cryo-EM map (unpublished) and the 2.92 Å GroEL X-ray crystal structure (PDB ID 1XCK) model-calculated map, showing an optimal voxel size of 1.06 Å. **E**, the Fourier shell correlation curve for the 20S Proteosome refined cryo-EM map showing a resolution FSC_0.143_ = 2.74 Å. **F**, the Fourier shell correlation curve for the GroEL refined cryo-EM map, resulting in a resolution FSC_0.143_ = 2.94 Å. The ChET-Cp-52 (**A**) and 20S Proteasome (**C**) cryo-EM data sets were collected with the same FEI Titan Krios at 300 kV equipped with a Gatan K3 detector at the same nominal magnification. The HeLaT-Cp-52 (**B**) and GroEL (**D**) cryo-EM data sets were collected with the same FEI Titan Krios 300 kV equipped with a K2 detector at the same nominal magnification.
